# The relationship between social participation and quality of life in individuals with traumatic brain injury

**DOI:** 10.20407/fmj.2024-016

**Published:** 2025-04-17

**Authors:** Toshinori Watanabe, Megumi Suzuki, Kouji Yamada, Naoki Aizu, Kikuo Ota

**Affiliations:** 1 Graduate School of Health Sciences, Fujita Health University, Toyoake, Aichi, Japan; 2 Department of Rehabilitation Medicine, Fujita Health University, School of Medicine, Toyoake, Aichi, Japan

**Keywords:** Traumatic brain injury, Social participation, Quality of life (QOL)

## Abstract

**Objective:**

To elucidate the relationship between social participation and quality of life (QOL) in patients with traumatic brain injury.

**Methods:**

This study included 128 community-dwelling patients with head injuries (average age: 41.8 years; average time since injury: 3126 days). We employed the Community Integration Questionnaire (CIQ; scoring range 0–29), a disease-specific scale for head injury patients, along with the Quality of Life after Brain Injury (QOLIBRI; scoring range 0–100%). An adjusted nonlinear regression analysis was used to explore the relationships between the CIQ (total score and subscales: home integration, social integration, productivity) and QOLIBRI (total score and subscales).

**Results:**

A significant relationship was observed only between the Total CIQ and the Self subscale of the QOLIBRI (p=0.006). The Self subscale score of the QOLIBRI increased with the Total CIQ score up to 15, after which it plateaued. Additionally, a significant positive relationship was found between the Social Integration subscale of the CIQ and the Self subscale of the QOLIBRI (p=0.018). The QOLIBRI Self score increased with the CIQ Social Integration score up to 8, beyond which it remained stable. No associations were found between the CIQ’s Home Integration and Productivity subscales and the QOLIBRI scores.

**Conclusion:**

The findings indicate that for individuals with traumatic brain injury, an increase in social participation, particularly in social integration, correlates with an enhanced sense of self-satisfaction up to a certain point. However, beyond this level, further advances in social participation do not yield additional gains, suggesting that factors other than social participation play a role in enhancing QOL. This underscores the multifaceted nature of QOL in this context.

## Introduction

Traumatic brain injury (TBI) commonly results in a spectrum of after-effects, including physical symptoms such as paralysis, sensory disturbances, visual field defects, and cerebellar ataxia, as well as higher brain dysfunctions. Consequently, individuals with TBI may be physically capable of performing daily activities yet find it challenging to engage in societal participation.^1,2^ Social participation lacks a universally agreed-upon definition, encompassing a broad range of activities. The concept includes engagement in social networks and informal social participation, which refers to the types of civic participation evident in everyday habits and activities carried out in public spaces, such as gardening, eating, cooking, and cycling. Additionally, it extends to include volunteer activities.^3,4^ Research has demonstrated a link between social participation and health outcomes. For instance, a cohort study among elderly individuals showed a positive correlation between baseline social participation and both mental and physical health,^4^ suggesting that active social engagement is crucial for maintaining and enhancing health. Thus, fostering social participation is considered vital for leading a healthy life within the community.

It has been observed that social participation decreases after TBI compared to pre-injury levels.^5^ Approximately 40% of individuals with TBI experience restricted social participation, with emotional and social challenges often reported as contributing factors based on subjective experiences.^6^ Factors influencing post-TBI social participation include younger age, longer time since onset, and higher Functional Independence Measure (FIM) motor and cognitive scores, which correlate with improved social engagement.^7^ Additionally, being single at the time of injury, possessing a higher level of education, and employment status are linked with greater community integration^8^ and better physical health two years post-injury.^9^ Furthermore, about 90% of individuals with TBI who suffer from higher brain dysfunction are living at home one year after their injury, and 25% return to work.^10^ Consequently, the physical and cognitive impairments associated with TBI tend to reduce the scope of activities and interactions, diminishing productivity. Higher brain dysfunction, an often invisible disability, may be challenging for others to understand; however, targeted rehabilitation and support can enhance social participation. For accurately assessing social participation in people with TBI, it is recommended to employ TBI-specific indicators that consider the unique characteristics of the condition’s aftermath.^11^

A decrease in social participation can lead to diminished feelings of achievement, impacting self-esteem and mood, ultimately affecting the HRQOL (Health-Related Quality of Life) of individuals with TBI.

HRQOL is defined as “a measure that reflects the impact of a person’s health status and disease on their subjective well-being, covering aspects such as mental health, vitality, pain, and daily activities like work, housework, and social engagements,” and this measure is employed to gauge the effectiveness of interventions in medical settings.^12^ HRQOL is considered one of the patient-reported outcome measures (PROMs) and is used to assess a patient’s perception of their symptoms and functional status. It is an indicator that captures information from the subjective perspective of the patient, including details that cannot be ascertained through objective assessments of physical function or daily living activities. HRQOL is valuable not only for helping patients make informed decisions but also for aiding clinicians in developing treatment plans and promoting service improvements by comparing patient health status before and after interventions.^13^

HRQOL in individuals with TBI is frequently assessed using the Short Form-36 (SF-36). Additional instruments include the Quality of Life after Brain Injury (QOLIBRI), the European Brain Injury Questionnaire (EBIQ), the Child Health Questionnaire (CHQ), and the World Health Organization Quality of Life short version (WHOQOL-BREF).^11^ Factors reported to enhance the quality of life for those with TBI encompass pre-injury employment,^14,15^ educational background, and a subjective sense of independence.^14^ Conversely, social isolation^16^ is known to diminish quality of life. Cognitive impairment is also believed to be a significant factor.^17^ Despite these insights, there remains a scarcity of research on the relationship between social participation and health-related quality of life among TBI patients.^18–20^

The relationship between social participation and HRQOL in individuals with TBI has been explored using the SF-36, a comprehensive evaluation tool.^9,21,22^ However, there is limited research concerning the impact of social participation on disease-specific HRQOL in TBI populations. Specifically, few studies have examined how different aspects of the quality of life in people with TBI correlate with social participation, using tools that are tailored to evaluate the unique aspects of TBI.

The Community Integration Questionnaire (CIQ)^23^ is a disease-specific tool designed to assess community integration for individuals with TBI. Community integration encompasses integration into home life, social networks, employment, education, volunteer activities, and other forms of productive engagement.^23^ These elements are aligned with the concepts of activities and participation outlined in the International Classification of Functioning, Disability, and Health (ICF), which is established by the World Health Organization (WHO).^24^

CIQ measures the social participation of TBI patients through three subscales: family integration, social integration, and productive integration. These scales provide a quantitative assessment of an individual’s roles within the family, social involvement, and engagement in productive activities. It has been noted that the total CIQ score positively correlates with the QOLIBRI subscale of daily life and autonomy.^25^ However, given the complex structure of QOL, a more detailed analysis is warranted. Previous studies have employed various statistical methods such as correlation analysis, one-way analysis of variance, simple regression analysis, and multiple regression analysis to explore the consequences of QOL and the factors affecting it using QOLIBRI.^14–16,25–35^ However, no studies have yet reported the use of nonlinear regression analysis. Implementing nonlinear regression could provide detailed insights into the variations in QOL and the level of achievement relative to the degree of social participation. Such detailed analysis could be invaluable in devising strategies to enhance social participation among people with TBI, potentially leading to improved overall quality of life.

The aim of this study is to elucidate the impact of social participation on the QOL of individuals with TBI. Furthermore, this research will employ nonlinear regression analysis to examine this relationship in greater detail. It is hypothesized that social participation is positively associated with HRQOL among people with TBI.

## Subjects and Methods

### Design

A survey utilizing a questionnaire on social participation and QOL was conducted among individuals with head injuries living in the community. Participants were recruited from 2013 to 2016. Medical staff at cooperating facilities were asked to identify eligible participants and distribute the questionnaires to them. Participants completed the questionnaires at home and returned them to the researchers via mail.

This study was approved by the Ethics Review Committee of Fujita Health University (approval number 11–167), and informed consent was obtained from all subjects prior to their participation.

### Subjects

The study involved 136 individuals living in the area who had suffered a head injury. These participants were recruited from local hospitals, rehabilitation centers, and centers for supporting people with higher brain dysfunction. Medical personnel at these facilities, who were cooperating with the study, gathered the necessary patient information from medical records.

The participants met the following selection criteria: They provided written informed consent voluntarily after receiving a thorough explanation about the study, ensuring they understood its details and purposes. Participants were between 16 and 79 years old at the time of consent, understood the purpose and methods of the examination, and were diagnosed with TBI based on the ICD-10 classification. Data from the Glasgow Coma Scale was available within 24 hours of the injury, and the Glasgow Outcome Scale Extended (GOSE) score was 3 or higher. More than three months had passed since the injury, the participants were 15 years or older at the time of the injury, and they had been discharged from the hospital.

### Evaluation Method

Community Integration Questionnaire (CIQ): The CIQ is a self-administered questionnaire designed to assess the social disadvantage or the degree of social participation among individuals with TBI.^23^ It includes questions covering the sub-scales of home integration (HI), social integration (SI), and productivity (PS). The questions address activities such as housework, shopping, leisure activities, visiting friends, and working hours. The questionnaire consists of 15 items, and the total score (Total CIQ) ranges from 0 to 29, with a higher score indicating a greater degree of social participation.

Regarding the breakdown of the subscale questions and scores in the evaluation, the HI subscale consists of 5 questions, with scores ranging from 0 to 10 points, focused on housework and shopping activities. The SI subscale includes 6 questions, scoring between 0 and 12 points, and covers leisure activities, visiting friends, and similar social interactions. The PS encompasses 4 questions, with scores ranging from 0 to 7 points, related to work, school, and volunteer activities. Details of these evaluation items are presented in Table 1 in the Appendix.^36^

Glasgow Coma Scale (GCS): The GCS is a clinical scale used to evaluate the level of consciousness and the depth and duration of impaired consciousness and coma. It assesses three aspects of behavior: motor response, verbal response, and eye movement, each scored individually.^37^ The total score for these three items ranges from 3 to 15, with a higher score indicating a better level of consciousness. Based on the GCS score, TBI is classified into three severity levels: a score of 3–8 indicates severe TBI, 9–12 indicates moderate TBI, and 13–15 is considered mild TBI.

Glasgow Outcome Scale-Extended (GOSE): The GOSE is used to is used to evaluate the severity and predict the prognosis after TBI.^38^ It is scored on a scale from 1 to 8, where higher scores denote better recovery outcomes. Specifically, scores of 3–4 indicate a severe injury, 5–6 suggest a moderate injury, and 7–8 represent good recovery.

Hospital Anxiety and Depression Scale (HADS): Developed by Zigmond in 1983,^39^ the HADS is a self-assessment tool designed to measure levels of anxiety and depression. It consists of 14 items, divided equally with 7 items each for anxiety and depression. Each item is scored on a 4-point scale ranging from 0 to 3, leading to a total score range from 0 to 21 for each of anxiety and depression. A higher score indicates more severe symptoms. The cutoff value for concern in either domain is 7 points.^40^

QOLIBRI (Quality of Life after Brain Injury; QOLIBRI): The QOLIBRI serves as the HRQOL scale for evaluating outcomes in this study. It is a self-administered questionnaire specifically designed to assess health-related quality of life in individuals with TBI.^41^ The questionnaire is segmented into six subscales, which include cognition, self, daily life and autonomy, social relationships, emotions, and physical problems. Responses are given on a 5-point Likert scale with options ranging from “not at all,” “slightly,” “moderately,” “quite a bit,” to “very much.” The total score, termed the Total QOLIBRI, is computed as a percentage from 0 to 100, where a higher score reflects a better quality of life. The reliability and validity of the Japanese version of the QOLIBRI have been confirmed by Suzuki et al.^42^ The detailed assessment items are listed in Table 2 in the Appendix.^33^

### Social Background and Clinical Characteristics

Information was collected from participants using a questionnaire that covered a range of topics. This included gender, age, and marital status, which was categorized as single, with a partner (spouse or lover), or without a partner (widowed or separated). Additional information gathered included the number of years of education, employment status, and living environment. Participants also assessed their subjective degree of independence, which ranged from completely independent to not at all independent, and their subjective sense of health, classified as either healthy or unhealthy.

Medical staff at the facility collected data from the medical records of participants, which included the number of days since the injury (calculated from the date of injury to the date of questionnaire completion), the GOSE, and the site of brain injury. Additionally, information on the presence or absence of disabilities was recorded, such as epilepsy, paralysis, visual impairment, hearing impairment, other traumas, communication disorders, attention disorders, memory disorders, executive function disorders, and emotional and behavioral disorders. It was also noted whether rehabilitation had been carried out. The degree of independence in daily life was assessed through a five-point Visual Analogue Scale. This scale evaluated various aspects of daily life including making phone calls, doing the laundry, and other household chores; necessary actions such as using the toilet, bathing, changing clothes, and eating; mobility aspects like using public transport and traveling; and management tasks such as paying bills, dealing with official notices, and keeping appointments. The scale ranged from 1, indicating no help needed, to 5, indicating always needs help.

### Analysis Method

For the analysis, the software IBM SPSS 23 (IBM Corp, Armonk, NY, USA) and R Studio (ver. 4.3.1) were utilized.

To assess the distribution of the CIQ scores in relation to the sociodemographic variables and clinical characteristics of the participants, the CIQ scores were categorized into three groups: 0–9, 10–19, and 20–29 points. The Chi-square test was employed to analyze these groupings. The association between the CIQ items and the subscales of QOLIBRI was examined using nonlinear regression analysis. A significance level of p<0.05 was applied in all cases.

## Results

Out of 136 respondents to the questionnaire, 8 were excluded due to many missing values, resulting in 128 participants for the study, comprising 103 men and 25 women. The sociodemographic and health characteristics of these participants were as follows: The average age of participants was 41.8 years with a standard deviation of ±14.2. Regarding marital status, 61 were single, 56 were with a partner, and 11 were without a partner. The average number of years of education was 13.2 with a standard deviation of 2.4. In terms of employment status, 46 were unemployed, 72 were employed, and 3 were students. The living environments reported included 18 living independently at home, 60 with assistance at home, 36 without assistance, 2 in supported housing, 8 in other living situations, and 1 did not respond. Participants also rated their subjective level of independence with 16 feeling completely independent, 47 mostly independent, 43 somewhat independent, and 22 not independent at all. The subjective sense of health showed 80 participants feeling healthy and 47 feeling unhealthy, while specific health conditions noted included 38 finding it difficult to see and 52 reporting a lack of physical strength.

The participants’ clinical data revealed the following:

The average number of days since the injury was 3126.4, with a standard deviation of ±2586 days. The GCS score, representing the worst value within 24 hours of injury, averaged 6.09 with a standard deviation of ±3.64. The GOSE score averaged 4.88, with a standard deviation of ±1.468. Locations of brain injury were distributed as follows: no image available for 9 participants, frontal lobe injuries in 43, temporal lobe injuries in 3, diffuse axonal injuries in 56, occipital lobe injuries in 7, and 10 participants did not respond. Reported disabilities included epilepsy in 31 participants, hemiplegia in 32, visual impairments in 22, hearing impairments in 6, non-traumatic brain injury related issues in 58, communication disorders in 76, attention disorders in 105, memory impairments in 94, executive function disorders in 93, and emotional and behavioral disorders in 74. The average scores for independence in daily life activities were as follows: daily activities 2.0±1.4, necessary movements 1.4±1.0, mobility 2.3±1.6, and management 2.9±1.6. Regarding rehabilitation, 48 participants were currently receiving rehabilitation services, and 104 had previously received them.

The average values and standard deviations for the QOLIBRI are as follows: Total QOLIBRI 42.5±19.0, Cognition 32.2±20.5, Self 32.5±22.6, Daily Life and Autonomy 37.3±24.8, Social Relationships 43.7±22.8, Emotions 61.5±26.0, and Physical Problems 57.9±25.6. For the CIQ, the average, standard deviation, and median values are: Total CIQ 13.4, 5.5, 13.0; HI scores were 2.9, 3.0, 2.25; SI scores were 6.2, 2.5, 6.0; PS scores were 4.3, 2.0, 13.0. HADS scores are: Anxiety 6.8±4.3 and Depression 8.6±4.9.

The CIQ scores for participants were divided into three groups, each spanning 10 points: 0–9 points, 10–19 points, and 20–29 points. The sociodemographic and clinical characteristics associated with these CIQ score ranges are detailed in Tables 1-1 and 1-2.

The distribution of CIQ scores among participants varied significantly across several sociodemographic and clinical characteristics. Notably, differences were observed in terms of employment status, GOSE scores, HADS scores for anxiety and depression, presence of hemiparesis, and participants’ sense of independence. To further analyze these relationships, a nonlinear regression analysis was conducted. This analysis adjusted for factors such as age, gender, education, employment, and marital status, which are thought to influence social participation.^5,43–46^

**Figure 1:**
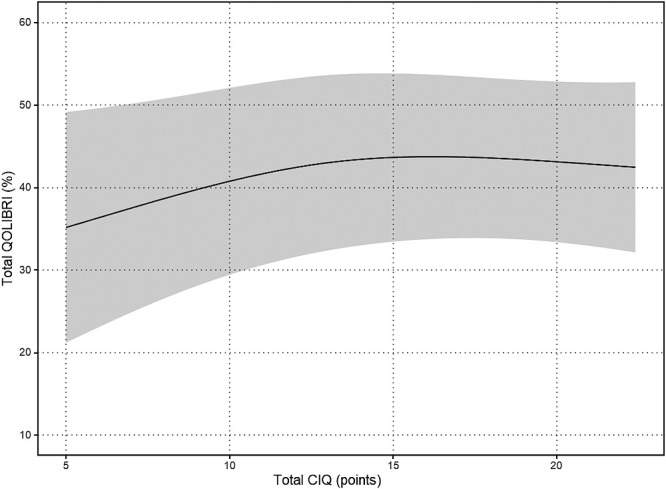
Relationship Between Total CIQ and Total QOLIBRI Nonlinear regression analysis (p=0.154) Total CIQ: Community Integration Questionnaire Total Score Total QOLIBRI: Quality of Life after Brain Injury Total Score

**Figure 5 F5:**
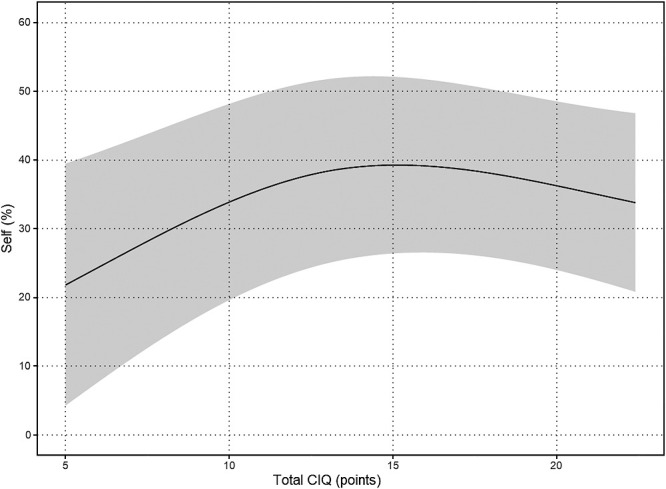
Relationship Between Total CIQ and Self Nonlinear regression analysis (p=0.006) Total CIQ: Community Integration Questionnaire Total Score

**Figure 6:**
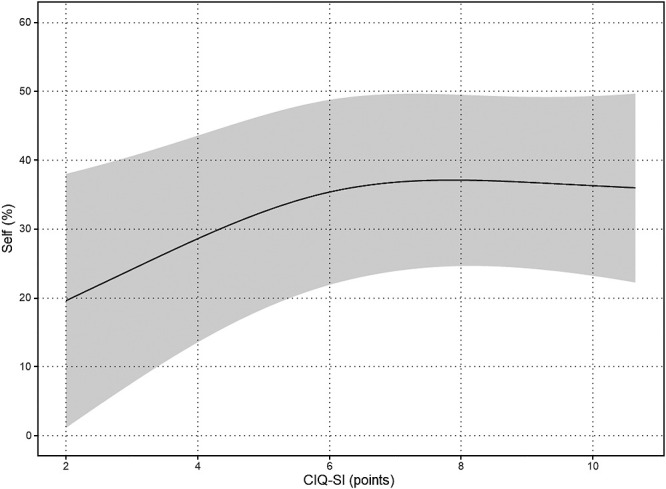
Relationship Between SI and Sel Nonlinear regression analysis (p=0.018) SI: Social Integration Scale

The results of the nonlinear regression analysis between the CIQ and the QOLIBRI are presented in Table 2 and Figures 1 [Fig F2][Fig F3][Fig F4][Fig F5]to 6. Specifically, a significant relationship was found only between the Total CIQ and the QOLIBRI Self subscale, with a p-value of 0.006, as shown in Table 2-1. The QOLIBRI Self score increases with the Total CIQ score up to 15 points, after which it plateaus, as depicted in Figure 5. In the HI scale, no significant association was detected between the Total QOLIBRI and its subscales, detailed in Table 2-2. Conversely, the SI scale showed a significant positive association between the SI and the QOLIBRI Self score (p=0.018), which is outlined in Table 2-3 and visually represented in Figure 6. For SI scores up to 8, the QOLIBRI Self score increased, but no further changes were observed beyond this point. No significant associations were found between the Total QOLIBRI and other subscales in the PS, as indicated in Table 2-4. Additional findings from other nonlinear regression analyses are illustrated in Figures 1 through 4 in the Appendix.

## Discussion

This study explored in detail the relationship between social participation and quality of life among people with TBI. The participants were mostly in their 40s, with a significant majority being male (80%) and about half (46%) being single. Additionally, the time since injury was noted to be long. These characteristics align with those found in previous epidemiological studies on TBI populations, suggesting that the study sample adequately represents the current state of individuals with TBI.^15^ Notably, 56% of participants were employed, and a significant majority (83.5%) had attained at least a high school education, indicating 12 or more years of schooling. Approximately 82.5% of participants were able to live with minimal assistance from others. The severity of injuries among participants was considerable, with GOSE scores indicating severe to moderate recovery levels. Furthermore, over 89.1% of participants exhibited some form of higher brain dysfunction. These findings suggest that while many participants managed daily life activities independently, cognitive impairments persisted, and full social reintegration had not yet been achieved.

This study revealed that while the Total CIQ score was higher than observed in previous research,^25^ the QOLIBRI Self score plateaued once the Total CIQ score reached 15 points. This indicates that QOL improves with increased social participation up to a certain threshold, beyond which further social engagement does not contribute to additional QOL improvements. In the realm of TBI, high mobility has been linked to enhanced social participation and better QOL.^47^ Despite 25% of participants in this study experiencing hemiplegia, most were generally able to move around independently in their daily lives. This capability likely facilitated their ability to engage socially, thereby enhancing their self-satisfaction to a certain degree. Previous studies have noted a positive correlation between Total CIQ and aspects of QOLIBRI related to daily life and autonomy,^25^ indicating that active participation in society may bolster one’s satisfaction with their ability to manage daily activities, although this relationship was not statistically significant in the current study. Contrarily, research by Burleigh et al. highlighted the absence of a linear correlation between Total CIQ and overall life satisfaction,^48^ supporting the findings from this study that the relationship between social participation and QOL in TBI is not straightforwardly linear. Up to a total CIQ score of 15, improvements were observed in the QOLIBRI self-score. However, once the total CIQ score reached 15, the QOLIBRI self-score showed minimal to no further change, suggesting that a CIQ score of 15 might represent a threshold beyond which further improvements in self-satisfaction do not occur. This phenomenon underscores that while social participation is integral to enhancing QOL to a certain extent, surpassing a certain level of social engagement does not influence QOL. It reflects the multifaceted nature of QOL, which is influenced by a variety of factors beyond social participation, such as employment status prior to injury,^14,15^ educational background, subjective independence,^14^ and the impact of social isolation.^16^ This complexity illustrates that improving the QOL of individuals with TBI requires a multidimensional approach that considers various life aspects.

In this study, no significant relationship was found between the CIQ-HI scores and the Total QOLIBRI scores or its subscales. The ability to perform household chores, as measured by the CIQ-HI, indicates a retained level of physical and mental function as well as a degree of independence in daily life.^25,36^ It also suggests a person’s capability to fulfill certain roles within the family, which is considered the smallest social unit. Despite these capabilities, it appears that social participation within the family setting alone is not sufficient to enhance QOL. With regard to family participation, it has been noted that the role an individual played in their family prior to an illness, such as being a housewife, and whether they had a spouse who participated in household duties, could influence their engagement level post-illness. This variation in roles could potentially lead to a decrease in their participation scores.^36^ It is suggested that providing support tailored to an individual’s pre-illness role within the family and considering the existing family structure could help improve their QOL.

In analyzing the CIQ-SI, it was observed that improvements in the QOLIBRI self-score continued up to a score of 8 points, highlighting a connection between social integration and a certain degree of self-satisfaction. This CIQ-SI score of 8 points, which falls within the average range, appears to represent an upper threshold for how much social integration can influence improvements in quality of life. This finding suggests that while social integration positively impacts quality of life for individuals with TBI up to a certain point, exceeding this level of social integration does not yield further improvements in quality of life, indicating that factors other than social integration also play significant roles. At TBI, numerous studies have highlighted the adverse effects of social isolation on QOL.^16^ Additionally, there is well-documented evidence showing a positive correlation between social integration and life satisfaction, as measured by the CIQ.^48^ Participants in this study demonstrated a relatively high level of independence in ADL, with about 60% actively engaging in leisure activities. Despite these engagements, they frequently ventured out, interacting with individuals outside their immediate family circle multiple times a week. It has been reported that TBI can lead to several cognitive and physical impairments, including mental fatigue, decreased motor ability, memory impairment, and executive function disorders. These issues are known to complicate everyday activities such as shopping and using public transportation.^49^ This study observed that many participants with higher brain dysfunction faced challenges in engaging in social activities such as leisure outings or shopping. However, the opportunity for these individuals to interact with people outside their family circles appeared to enhance their personal satisfaction. This finding suggests that, despite the presence of higher brain dysfunction, QOL can be improved by providing support that facilitates community involvement and social interaction with friends and acquaintances. Research has shown that individuals who engage in programs designed to promote social and recreational activities tend to exhibit higher levels of social participation.^50^ Such programs not only enhance social participation but also increase personal satisfaction and reduce the caregiving burden on families, particularly for those with acquired brain injuries averaging 7.7 years post-injury.^51^ Consequently, it is crucial to provide support that encourages social interaction and participation in leisure activities for people with TBI, especially those scoring between 0 and 8 on the CIQ-SI. Facilitating these opportunities can improve their social integration and overall quality of life.

In the analysis of the CIQ-PS, there was no significant association found between Total QOLIBRI scores and its subscales. Previous research has highlighted that participation in productive activities can significantly impact the physical and mental health of individuals with TBI, even up to 20 years post-injury. Factors such as pre-injury employment,^14,15,22^ high educational attainment, a strong sense of subjective independence,^14^ and active participation in work have all been associated with higher QOL.^52^

In this study, the analysis of CIQ scores in relation to sociodemographic variables and clinical characteristics revealed that employed individuals tended to be more socially engaged, suggesting that employment facilitates social participation. However, productive activities, typically associated with employment, were not found to correlate directly with QOL. Previous research has indicated a relationship between productivity and aspects of QOL, specifically the “Daily life and autonomy” subscale of the QOLIBRI.^25^ However, those studies did not specify the employment status of their participants, making direct comparisons challenging. In this study, the SI score was notably higher (average 6.2 points) compared to an average of 2 points reported in earlier studies. With over half of the participants in this study being employed, and observing a higher level of social integration than seen in previous research, it raises the possibility that employment-related issues themselves may influence QOL.

Productivity is considered vital for improving the physical and mental health quality of life for individuals with TBI.^22^ However, studies suggest that part-time employment, rather than full-time work, is more likely to meet the needs of individuals with TBI and facilitate their social integration.^53^ This observation implies that the stress associated with long working hours and the resultant limited time available for social interactions might not contribute positively to quality of life improvements, despite the individuals being employed. To optimize integration into the local community and ensure sustainable labor participation from a long-term perspective, it’s essential to provide vocational rehabilitation and employment support,^54,55^ and to consider environmental factors.^56^ This includes providing detailed information to the workplace where the individual is returning and offering ongoing support after they have returned to work. These measures are crucial not only for facilitating the initial employment process but also for ensuring that the individual can maintain their employment over time.

Long-term predictors of community reintegration following TBI have been identified as the age at which the injury occurred, cognitive function, and physical pain.^57^ It has been reported that comprehensive and integrated neuropsychological rehabilitation can enhance community reintegration, functional independence, and productivity.^58^ Additionally, intensive cognitive rehabilitation has been found beneficial in improving social participation, and it is associated with increased satisfaction with social involvement and higher cognitive levels.^59^ Given these findings, there is a strong case for focusing on cognitive function in rehabilitation, starting from the acute stage of TBI, to achieve better outcomes in various aspects of community integration such as performing household chores, engaging in leisure activities, socializing with friends, and maintaining employment. Essentially, initiating cognitive rehabilitation early in the treatment process can foster greater social participation and enhance the overall quality of life for individuals with TBI.

In this study, the Total CIQ and SI scores were found to correlate with the self of the QOLIBRI scale. It was observed that the ability to go out and engage in social interactions is somewhat related to enhancing self-satisfaction among individuals with TBI. This relationship does not follow a linear trend as suggested by previous research;^25^ instead, by employing nonlinear regression analysis, we were able to more precisely capture changes in QOL in a curved manner. This approach allowed for a more accurate elucidation of the impact of social participation on QOL. The findings indicate that the effects of social participation on QOL are limited, and there are aspects of QOL that are not improved by social participation alone. Therefore, a comprehensive rehabilitation and support strategy that takes into account environmental factors, pre-morbid social background, and individual characteristics of people with TBI is advocated. It is believed that enhancing social acceptance to prevent loneliness and stabilizing the support system for productive activities and personal life could lead to further improvements in quality of life. This approach highlights the need for multi-faceted support systems to effectively enhance the overall well-being of individuals with TBI.

## Figures and Tables

**Figure 2 F2:**
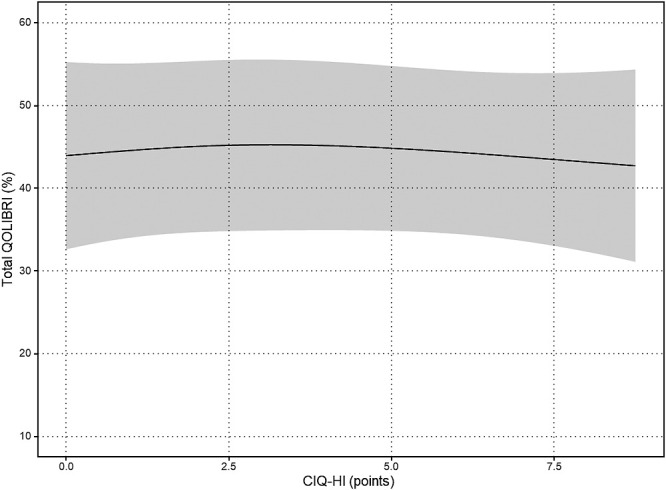
Relationship Between HI and Total QOLIBRI Nonlinear regression analysis (p=0.859) HI: Home Integration Scale Total QOLIBRI: Quality of Life after Brain Injury Total Score

**Figure 3 F3:**
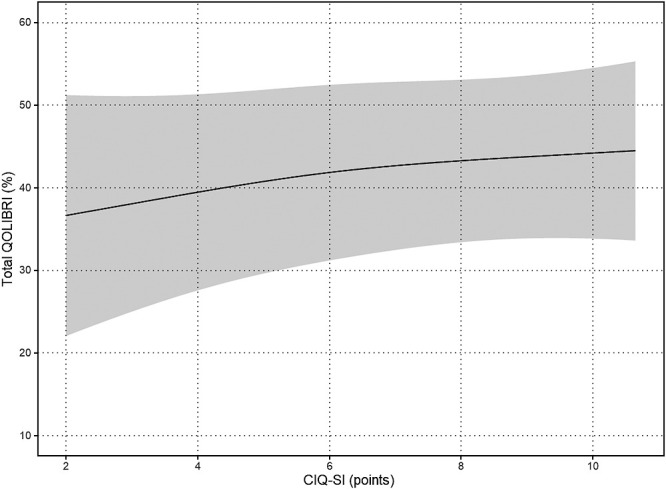
Relationship Between SI and Total QOLIBRI Nonlinear regression analysis (p=0.362) SI: Social Integration Scale Total QOLIBRI: Quality of Life after Brain Injury Total Score

**Figure 4 F4:**
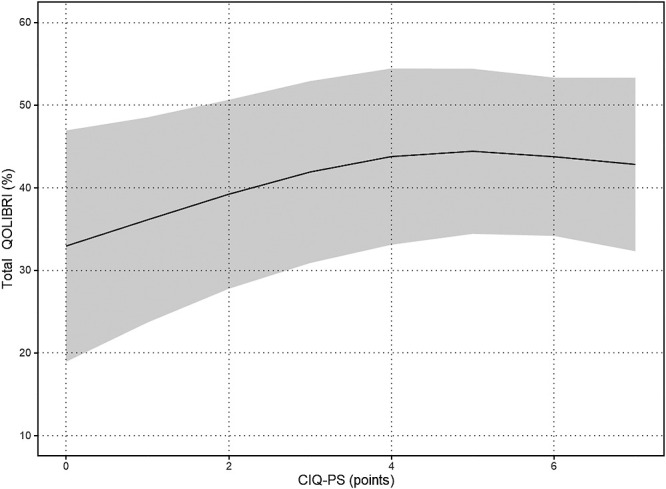
Relationship Between PS and Total QOLIBRI Nonlinear regression analysis (p=0.084) PS: Productivity Scale Total QOLIBRI: Total Score for Quality of Life after Brain Injury

**Table 1-1:** Sociodemographic Characteristics by CIQ Score

Variable	CIQ score			p-value
	0~9 (n=31)	10~19 (n=75)	20~29 (n=22)	
Age (years)	41 (36, 60)	40 (31, 52)	38 (27, 46)	0.182
Sex (people)				0.424
Female	4 (12.9%)	15 (20.0%)	6 (27.3%)	
Male	27 (87.1%)	60 (80.0%)	16 (72.7%)	
Marital Status (people)				0.13
with partner	14 (45.2%)	34 (45.3%)	8 (36.4%)	
separated from partner	1 (3.2%)	5 (6.7%)	5 (22.7%)	
Single	16 (51.6%)	36 (48.0%)	9 (40.9%)	
education years				0.591
9	5 (15.2%)	8 (10.7%)	3 (15.0%)	
12	18 (54.5%)	28 (37.3%)	8 (40.0%)	
15–16	9 (27.3%)	36 (48.0%)	8 (40.0%)	
others	1 (3.0%)	3 (4.0%)	1 (3.9%)	
Employment Status (people)				**<0.001**
Employed	9 (30.0%)	45 (61.6%)	18 (81.8%)	
Not employed	21 (70.0%)	28 (38.4%)	4 (18.2%)	

* Bold means p-value p<0.05. Pearson’s chi-square test, Kruskal-Wallis test The brackets for age indicate the 25% and 75% percentiles of the variable’s quartile range in the three groups of CIQ scores: 0–9 points, 10–19 points, and 20–29 points. CIQ: Community Integration Questionnaire

**Table 1-2:** Sociodemographic and Clinical Characteristics by CIQ Score

Variable	CIQ score			p-value
	0~9 (n=31)	10~19(n=75)	20~29 (n=22)	
Major Lesion of Brain (people)				0.651
diffuse	16 (59.3%)	31 (43.7%)	9 (45.0%)	
none on image	0 (0.0%)	7 (9.9%)	2 (10.0%)	
occipital lobe	2 (7.4%)	4 (5.6%)	1 (5.0%)	
frontal lobe	9 (33.3%)	26 (36.6%)	8 (40.0%)	
temporal lobe	0 (0.0%)	3 (4.2%)	0 (0.0%)	
GOSE score (points)	3.0 (3.0, 4.0)	5.0 (4.0, 6.0)	6.0 (5.2, 6.0)	**<0.001**
HADS Anxiety (points)	6.0 (4.0, 10.0)	6.0 (3.0, 9.0)	9.0 (7.0, 10.0)	**0.022**
HADS Depression (points)	12.0 (7.5, 15.0)	7.0 (3.0, 11.0)	8.0 (5.5, 10.8)	**<0.001**
Self-reported independent status (people)				**<0.001**
Completely independent	1 (3.2%)	11 (14.7%)	4 (18.2%)	
Mostly independent	3 (9.7%)	31 (41.3%)	13 (59.1%)	
A little bit independent	12 (38.7%)	27 (36.0%)	4 (18.2%)	
Not independent at all	15 (48.4%)	6 (8.0%)	1 (4.5%)	
Self-reported health status (people)				0.138
Healthy	15 (48.4%)	51 (68.9%)	14 (63.6%)	
Unhealthy	16 (51.6%)	23 (31.1%)	8 (36.4%)	
Rehabilitation (people)				
previously	28 (90.3%)	60 (80.0%)	16 (72.7%)	0.246
Actually	14 (45.2%)	28 (37.3%)	6 (27.3%)	0.415
Clinical Symptoms (people)				
epilepsy	6 (19.4%)	19 (25.3%)	6 (27.3%)	0.755
hemiparesis	12 (38.7%)	18 (24.0%)	2 (9.1%)	**0.047**
visual deficit	11 (35.5%)	10 (13.3%)	1 (4.5%)	**0.005**
auditory deficit	2 (6.5%)	2 (2.7%)	2 (9.1%)	0.395
extra injury	18 (58.1%)	35 (46.7%)	5 (22.7%)	**0.037**
communication disorder	25 (80.6%)	39 (52.0%)	12 (54.5%)	**0.021**
attention disorder	27 (87.1%)	60 (80.0%)	18 (81.8%)	0.687
memory disorder	29 (93.5%)	52 (69.3%)	13 (59.1%)	**0.009**
executive function disorder	29 (93.5%)	50 (66.7%)	14 (63.6%)	**0.011**
affective and behavior disorder	24 (77.4%)	39 (52.0%)	11 (50.0%)	**0.039**

* Bold means p-value p<0.05 Pearson’s chi-square test, Kruskal-Wallis test The brackets in GOSE and HADS indicate the 25% and 75% percentiles of the variable’s quartile range for the three groups of CIQ scores: 0–9, 10–19, and 20–29. CIQ: Community Integration Questionnaire GOSE: Glasgow Outcome Scale-Extended HADS: Hospital Anxiety and Depression Scale

**Table 2-1:**
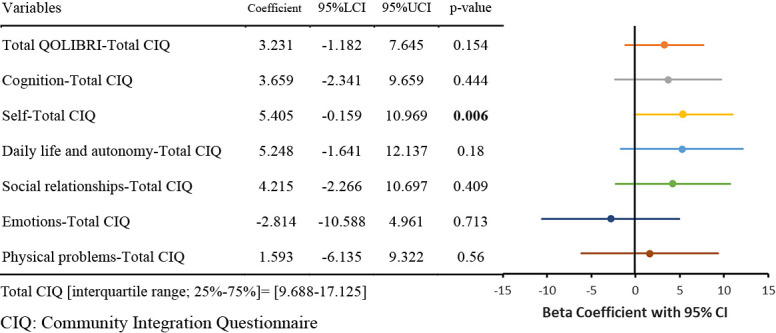
Relationship Between Total CIQ and QOLIBRI

**Table 2-2:**
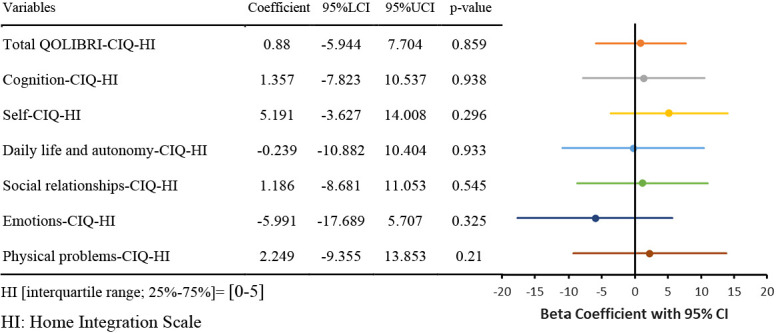
Relationship Between HI and QOLIBRI

**Table 2-3:**
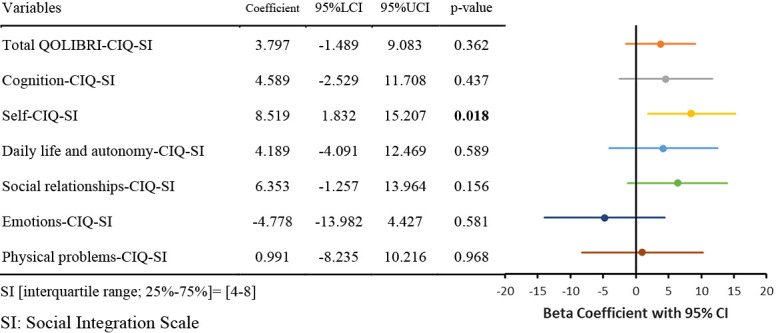
Relationship Between SI and QOLIBRI

**Table 2-4:**
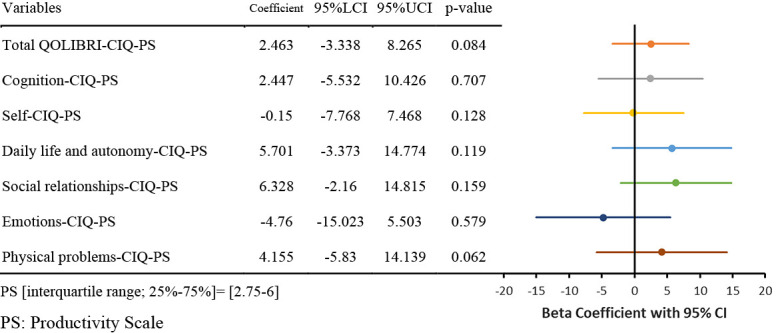
Relationship Between PS and QOLIBRI

Nonlinear regression analysis, with p-values in bold indicating p<0.05. CIQ: Community Integration Questionnaire HI: Home Integration Scale SI: Social Integration Scale PS: Productivity Scale QOLIBRI: Quality of Life after Brain Injury The Beta coefficients from the nonlinear regression analysis of the CIQ and QOLIBRI are displayed in the forest plot. The solid colored lines represent the 95% confidence intervals of the predicted values, with the central point of the solid line indicating the Beta coefficient. LCI: Lower Confidence Interval UCI: Upper Confidence Interval CI: Confidence Interval

## Limitations of the Study

This study faced several limitations, notably the inability to analyze specific factors such as the roles of family members prior to the injury, the characteristics of fatigue and stress associated with employment, and the influence of the specific sites of brain damage on outcomes.

Looking ahead, further research will explore whether improvements in HRQOL can be observed in individuals with TBI who experience changes in their social participation due to rehabilitation, community, or medical support. This future research aims to provide a more comprehensive understanding of the factors contributing to recovery and integration for people with TBI.

## Supplementary Material

PDF-Japanese
