# Supplementary material for: The relationship between social participation and quality of life in individuals with traumatic brain injury
Source: Fujita Med J. 2025 Apr 17;11(3):111–20. doi: 10.20407/fmj.2024-016 (PMC12327209; doi:10.20407/fmj.2024-016)
Supplement: Supplementary file 1 — PDF-Japanese [file fmj-11-111-s001.pdf]

タイトル：
外傷性脳損傷者の社会参加と生活の質（Quality of Life）の関連

ランニングタイトル：
外傷性脳損傷者の社会参加と生活の質の関連

Toshinori Watanabe, MSc[1)]
Megumi Suzuki, PhD[1)]
Kouji Yamada, PhD[1)]
Naoki Aizu, PhD[1)]
Kikuo Ota, M.D.,PhD[2)]

1） Graduate School of Health Sciences, Fujita Health University, Toyoake, Aichi, Japan

2） Department of Rehabilitation Medicine, School of Medicine, Fujita Health University, Toyoake, Aichi, Japan

Type of article: Original Article

Corresponding author:

Professor Megumi Suzuki, PhD
Graduate School of Health Sciences, Fujita Health University,
1-98, Dengakugakubo, Kutsukake-cho, Toyoake, Aichi 470-1192, Japan
Phone: +81-562-93-9000
Fax: +81-562-93-6817
E-mail: suzume@fujita-hu.ac.jp

抄録：

目的：外傷性脳損傷者の社会参加と Quality of Life(QOL)の関連を明らかにすることである。

方法 : 地域在住の頭部外傷者 128 名（平均年齢 41.8 歳、受傷後平均日数 3126 日）を対象とした。頭部外傷者の疾患特異的尺度である Community Integration Questionnaire(CIQ; 0-29 点)と Quality of Life after Brain Injury(QOLIBRI; 0-100%)を使用した。CIQ(総得点と下位尺度; 家庭統合・社会統合・生産性)と QOLIBRI（総得点と下位尺度）の関連を調べるのに調整された非線形回帰分析を用いた。

結果： Total CIQ は QOLIBRI 下位尺度 Self のみに有意な関連が認められた(p=0.006)。Total CIQ 15 点までは QOLIBRI の Self は上昇し、それ以降はほぼ変化がなかった。また、CIQ 下位尺度の社会統合と QOLIBRI の Self に有意な正の関連が認められた(p=0.018)。社会統合 8 点までは QOLIBRI の Self は増加し、それ以降はほぼ変化はみられなかった。CIQ の家庭統合と生産性に QOLIBRI との関連は認めなかった。

考察：外傷性脳損傷者の社会参加で特に社会統合が進むと自分自身への満足度が一定の程度まで上昇することが示唆された。一方で、たとえ社会参加が進んだとしても一定のレベル以上の値の増加がみられなかったことから、QOL の向上には社会参加のみでなく、それ以外の様々な要因が関わることが示唆され、QOL のもつ多面性が明らかになったと考えられた。

キーワード : 外傷性脳損傷、社会参加、QOL

はじめに

外傷性脳損傷（Traumatic Brain Injury：TBI）者の後遺症では、麻痺、感覚障害、視野欠損、小脳失調といった身体症状だけでなく、高次脳機能障害を伴うことが多い。そのため、たとえ日常生活活動が自立していても、社会参加に至らないことが多いとされる[1,2]。社会参加は一致した定義はなく、社会的なネットワークやインフォーマルな社会参加（公共の場での日常的な習慣や活動を通して観察される一種の市民参加；園芸、食事、料理、サイクリングなど）やボランティア活動を含む概念である[3,4]。社会参加と健康指標は関連を示すと報告され、高齢者を対象としたコホート研究では、ベースラインの社会参加が精神的および身体的健康と正の相関があったと報告されている[4]。つまり、社会参加をすることは、健康状態の維持や改善と関連する。社会参加を促進することは、地域生活を健康に過ごすために重要な視点であると考えられる。

TBI 後の社会参加は受傷前よりも低下することが報告されている[5]。TBI 者と社会参加の帰結については、TBI 者の 40%が社会参加は制限され、主観的な経験に基づく感情的な困難さや社会的な困難さが関連する[6]。TBI の社会参加に影響する因子について、年齢が若いこと、発症後年数が長いこと、FIM の運動項目・認知項目が高値であることが社会参加を良好にするすることや[7]、受傷時に独身であったこと、教育レベルが高い、就労していたことが、高い地域統合と関連すること[8]、TBI 者の受傷 2 年後の身体的健康に関連することが報告されている[9]。また、高次脳機能障害を有している TBI 者の約 90%は受傷後 1 年時に自宅で生活し、職業復帰した人の割合は 25%であった報告がある[10]。このように、TBI 者の身体的・認知的障害は活動範囲および交流範囲の狭小化、生産性低下することが予想される。高次脳機能障害は目に見えない障害であり周囲に理解されにくい特徴があるが、適切なリハビリテーションや対応をとることで、社会参加は促進される可能性がある。TBI 者の社会参加を表現するには、その後遺症の特徴を考慮して TBI 疾患特異的な指標を用いることが望ましい[11]。

社会参加の減少は達成感を低下させ、自己肯定感や気分に影響を与える可能性があり、TBI 者の健康関連の生活の質（health related quality of life; HRQOL）を低下させると推測される。

HRQOL は、「健康状態や疾患が、患者の主観的健康感（メンタルヘルス、活力、痛みなど）や日常生活（仕事、家事、社会活動）にどのような影響を与えているかを定量化したもの」で医療現場において介入効果を測定する際に用いられる QOL と定義される[12]。HRQOL は患者が直接報告するアウトカム（patient reported outcome measures; PROMs）のひとつであり、症状、機能状態について、患者の見解を評価するのに用いられる。つまり、患者の主観的視点から得られる情報であり、客観的な身体機能や生活機能評価では得られない情報が多く含まれる指標である。患者がより適切な意思決定を行い、臨床医が治療方針をたてるのに役立つだけでなく、治療前後の患者の健康状態を比較することでサービスの改善を促進することができると言われる[13]。

TBI の HRQOL は Short Form-36(SF-36)で評価されることが多い。そのほかに Quality of Life after Brain Injury(QOLIBRI)、European Brain Injury Questionnaire (EBIQ)、Child Health Questionnaire (CHQ)、the World Health Organization Quality of Life short version (WHOQOL-BREF)が用いられる[11]。これまでに TBI 者の QOL を高くする因子は、病前の就労[14,15]、教育歴、主観的な自立度[14]で、低下の因子は社会的孤立[16]が報告されている。また、認知障害が重要な役割を果たしているとされる[17]。

しかし、TBI 者の社会参加と健康関連 QOL の関連についての報告は少ない[18-20]。

TBI 者の社会参加と HRQOL の関係は包括的な評価ツールである SF−36 を用いてこれまで報告されたが[9, 21, 22]、社会参加が TBI 者の疾患特異的 HRQOL に関連するのかについての報告は少ない。特に、TBI 者の QOL の様々な側面と社会参加との関係を TBI の疾患特異的な評価を用いて調査したものは少ない。

Community Integration Questionnaire(CIQ)[23] は TBI の地域統合を評価する疾患特異的な質問紙である。地域統合は、家庭への統合、社会的なネットワークへの統合、雇用、学校、ボランティア活動などの生産活動への統合として定義される[23]。これらは、世界保健機関（World Health Organization）の定める国際生活機能分類(International Classification of Functioning, Disability and Health)[24] の活動と参加に含まれる概念である。

CIQ で表現される TBI 者の社会参加は、家庭統合、社会統合、生産統合であり、家庭内での役割、社会との関り、生産的活動について、定量的に評価するものである。CIQ の総得点が QOLIBRI の下位尺度の日常生活（Daily life and autonomy）と正の相関があることが報告された[25]が、QOL の多面的構造を考慮すると、従来よりも詳細な解析が望ましい。これまで QOLIBRI を用いた QOL の帰結や QOL に影響する因子について、相関分析や一元配置分散分析、単回帰分析、重回帰分析を用いて研究されてきたが[14-16, 25-35]、非線形回帰での分析は報告されていない。非線形回帰分析によって社会参加の程度による QOL の変動や到達点などの詳細な情報を得ることが期待でき、結果として TBI 者の社会参加を促進させる方策を考慮するのに利すると考えられる。

本研究の目的は、外傷性脳損傷者の社会参加が QOL に与える影響を明らかにすることである。また、非線形回帰分析によってより詳細な関連を明らかにすることである。TBI 者の社会参加は HRQOL と正の関連があると予測する。

対象と方法

デザイン

2013 年から 2016 年の間に募集した地域在住の頭部外傷者に対して社会参加と QOL に関する質問紙を用いた調査を実施した。研究者が協力施設の医療従事者に依頼し、参加可能な研究対象者の選出と、対象者へのアンケート配布をしてもらった。研究対象者は自宅でアンケートを記入後、郵送にて回答を研究者に返信して回収した。

本研究は、藤田医科大学倫理委員会の承認（承認番号 11-167）を得て実施し、対象者には事前にインフォームドコンセントを得た。

対象

研究対象者は、地域在住の頭部外傷者 136 名であった。対象者は、地域の病院、リハビリテーションセンター、高次脳機能障害の支援センターから募集された。協力者である施設勤務の医療従事者がカルテから医学的な患者情報を取得した。

対象者の選択基準は、1）本研究の参加にあたり、十分な説明を受けた後に、十分な理解の上に本人の自由意志による文書同意が得られた者、2）同意取得時に 16 歳以上 79 歳以下の者、3) 検査の実施の目的及び実施方法を理解している、4）ICD-10 分類により TBI と診断されている、5）

Glasgow Coma Scale の受傷後 24 時間以内のデータがある、6）Glasgow Outcome Scale Extended≧3 7) 受傷から 3 カ月以上経過している、8）受傷時の年齢が 15 歳以上である、9）退院している、を満たしているものとした。

評価方法

Community Integration Questionnaire(CIQ)：CIQ は TBI の社会的不利の状況、つまりは社会参加の程度を評価する質問紙で、対象者による自己記入式である [23]。質問は、家庭統合(Home integration scale；HI)と社会統合(Social integration scale；SI)、生産統合(Productivity scale；PS)の下位尺度について、家事、買い物、レジャー活動、友人訪問、就業時間について問う。アンケートの項目は 15 項目、総得点（Total CIQ）は 0-29 点で表現され、得点が高いほど社会参加の程度が高いことを意味する。

下位尺度の質問項目と得点の内訳について、HI は家事・買い物についての 5 項目（0-10 点）である。SI は、レジャー活動・友人訪問等についての 6 項目（0-12 点）である。PS は、就労・就学・ボランティア活動の 4 項目（0-7 点）である。評価項目を Appendix の table1 に示した [36]。

Glasgow Coma Scale(GCS)：意識レベルを評価するスケールである。意識障害と昏睡状態の深さと期間を評価する臨床尺度であり、行動の 3 つの側面（運動、言語、眼球運動）が個別に測定される [37]。3 つの項目の合計は 3-15 点で、得点が高いほど、意識レベルが良好であることを意味する。スコア 3–8 点：重度、9–12：中程度、13–15：軽度の TBI と分類される。

Glasgow Outcome Scale-Extended (GOSE）：TBI 後の重症度・予後予測の評価尺度である [38]。1-8 点で採点され、数値が大きいほど良好な回復を示す。スコア 3-4：重症、5-6：中等度、7-8：良好な回復と分類される。

Hospital Anxiety and Depression Scale（HADS）： Zigmond(1983) [39] によって開発された、不安、抑うつを測定する評価尺度である。不安について 7 項目、抑うつについて 7 項目の合計 14 項目からなる自己評価式の検査である。各項目 0-3 点の 4 段階で、不安と抑うつのそれぞれ合計 0-21 点で採点される。点数が高いほど、症状が重いことを意味する。カットオフ値が 7 点とされている [40]。

QOLIBRI (Quality of Life after Brain Injury；QOLIBRI; 脳損傷後の生活の質)

アウトカムとしての HRQOL 尺度には、QOLIBRI を使用した。QOLIBRI は TBI の健康関連の生活の質を評価する自己記入式の質問紙である [41]。思考力(Cognition)、自分自身(Self)、日常生活(Daily life and autonomy)、社会的関係(Social relationships)、感情(Emotions)、身体的問題(Physical problems)の 6 つの下位尺度に分かれている。質問項目は 5 ポイントのリッカート尺度で自己評価される（まったく、わずかに、中程度に、かなり、非常に）。総得点（Total QOLIBRI）は 0～100 パーセントで算出し、数値が高いほど良好な QOL を示す。Suzuki et al.によって、日本語版の信頼性と妥当性が検証された [42]。評価項目を Appendix の table2 に示した [33]。

社会背景と臨床的特徴

参加者より以下の情報を質問紙により取得した：性別、年齢、婚姻状況：独身、パートナー（配偶者や恋人）有り、パートナーなし（死別もしくは離別）、教育年数、就労状況、生活環境、主観的自立度（完全自立、ほぼ自立、少し自立、全く自立していない）、主観的健康感（健康、不健康）。

施設の医療従事者がカルテから以下の情報を収集した：受傷後日数（受傷日から質問紙記入日まで）、GOSE、脳損傷部位、障害（てんかん、麻痺、視覚障害、聴覚障害、その他の外傷、コミュニケーション障害、注意障害、記憶障害、遂行機能障害、情動・行動障害）の有無、リハビリテーションの有無。日常生活の自立度（日常活動; 電話する・洗濯・家事など、必要動作; トイレ・入浴・着替え・食事など、移動;公共交通機関の利用・旅行するなど、管理;支払い・役所からの通知書・約束など）は 1(手助け必要なし)から 5（いつも手助け必要）までの 5 段階の Visual Analogue Scale を用いた。

解析方法

ソフトウェア IBM SPSS 23 (IBM Corp, Armonk, NY, USA) と R studio（ver. 4.3.1）を解析に用いた.

社会人口学的変数と参加者の臨床的特徴による CIQ 得点の分布を調べるために、CIQ の点数（0-9、10-19、20-29 点）を 10 点ずつの 3 群に分け、Chi-square test を用いた。CIQ の項目と QOLIBRI の下位尺度の関連は非線形回帰分析で検証した。いずれの場合も $p<0.05$ を有意水準とした。

結果

136 名がアンケートに回答したが、欠損値が多い 8 名を除外し、128 名（男性 103, 女性 25）を対象者とした。社会人口学的変数の値（括弧内は人数分布）は次の通りであった。年齢（平均±標準偏差）41.8±14.2 歳、婚姻状況（独身 61, パートナーあり 56, パートナーなし 11）、教育年数（平均±標準偏差）13.2±2.4 年、就労状況（無職 46, 仕事あり 72、学生 3）、生活環境（(自宅で自立 18、自宅で介助あり 60、自宅で介助なし 36、介助付き住宅 2、その他 8、無回答 1)、主観的自立度（自立 16、ほぼ自立 47、少し自立 43、自立していない 22)、主観的健康感（健康 80、不健康 47)、健康状態（見えにくい 38、体力がない 52)。

参加者の臨床的特徴は、以下の通りであった。

受傷後日数 3126.4±2586 日、GCS（平均±標準偏差；受傷後 24 時間以内で最も悪い値）6.09±3.64、GOSE（平均±標準偏差）4.88±1.468、脳損傷部位（画像上なし 9, 前頭葉 43、側頭葉 3、びまん性軸索損傷 56、後頭葉 7、無回答 10）障害（てんかん 31、片麻痺 32、視覚欠損 22、聴覚障害 6、脳外傷以外の外傷 58、コミュケーション障害 76、注意障害 105、記憶障害 94、遂行機能障害 93、情動・行動障害 74)、日常生活の自立度（平均±標準偏差）[日常活動 2.0±1.4、必要動作 1.4±1.0、移動 2.3±1.6、管理 2.9±1.6]、リハビリテーション（現在受けている 48、以前は受けていた 104）

QOLIBRI の平均値±標準偏差は、Total QOLIBRI 42.5±19.0、cognition 32.2±20.5、self 32.5±22.6、daily life and autonomy 37.3±24.8、social relationships 43.7±22.8、emotions 61.5±26.0、physical problems 57.9±25.6 であった。CIQ の平均値、標準偏差、中央値は、Total CIQ 13.4、5.5、13.0、HI 2.9、3.0、2.25、SI 6.2、2.5、6.0、PS 4.3、2.0、13.0 であった。HADS（平均±標準偏差）は、不安 6.8±4.3、抑うつ 8.6±4.9 であった。

CIQ の点数を 10 点ずつの 3 群（0-9、10-19、20-29 点）に分け、CIQ の点数別の社会人口統計学的特性および臨床的特徴を Table1-1 と 1-2 に示した。

社会人口学的変数と参加者の臨床的特徴別の CIQ の点数による分布は、就労の有無、GOSE、HADS の不安、HADS の抑うつ、片麻痺（hemiparesis）、自立感の人数分布に有意差が認められた。これらの変数と、社会参加に関連するとされる年齢、性別、教育、就労、婚姻を調整因子[5,43-46]として非線形回帰分析を実施した。

＜Table1-1 の挿入位置＞＜Table1-2 の挿入位置＞

CIQ と QOLIBRI の非線形回帰分析の結果を、Table2、Figure1 から Figure6 に示した。Total CIQ と QOLIBRI の関連では、Total CIQ と QOLIBRI の Self のみに有意な関連が認められた(p=0.006)(Table2-1)。Total CIQ 15 点までは QOLIBRI の Self は増加し、それ以降はほぼ変化がなかった(Figure 5)。HI においては、Total QOLIBRI とその下位尺度で有意な関連はみられなかった(Table2-2)。SI においては、SI と QOLIBRI の Self のみに有意な正の関連が認められた(p=0.018)(Table2-3)(Figure 6)。SI 8 点までは QOLIBRI の Self のスコアは増加し、それ以降はほぼ変化はみられなかった。Total QOLIBRI とそのほかの下位尺度には関連が認められなかった。PS においては、Total QOLIBRI と下位尺度で有意な関連はみられなかった(Table2-4)。そのほかの非線形回帰分析の結果については、Appendix の Figure1 から Figure4 に示した。

＜Table2-1、2-2、2-3、2-4 の挿入位置＞
＜Figure1、2、3、4、5、6 の挿入位置＞

考察

この研究では、TBI 者の社会参加が QOL に関連することを詳細に検証した。参加者の平均年齢は 40 代、男性が多く（80%）独身が約半数(46%)で受傷後期間が長かった。これらは過去の TBI 者に関する疫学調査と同様の傾向であり、TBI 者の現状を表現していることが示唆された[15]。就労しているものが半数(56%)、学歴は高卒以上(12 年以上)が約 8 割（83.5%）、日常生活の自立度は約 8 割(82.5%)が自立から少しの介助で生活可能、受傷時の重症度は高く、GOSE は重度から中等度の回復レベルであった。何らかの高次脳機能障害を有しているものが 89.1%以上であった。これらのことから、研究参加者は身の回りのことは自分でできるが、認知機能障害があり、十分に社会的に回復していない対象者が多かったと推測された。

Total CIQ の点数は先行研究[25]よりも高いが、Total CIQ 15 点以降は QOLIBRI の Self はほぼ変化がなかった。つまり、ある程度までは社会参加が進むにつれ QOL は向上するが、それ以上では社会参加が進むだけでは QOL に変化がもたらされない可能性が示唆された。TBI において、高いレベルの移動能力は、高い社会参加と QOL に関連することが報告されている[47]。今回の参加者の 25％に片麻痺があったが日常生活における移動は概ね可能であった。そのため、ほとんどの参加者が外出による社会参加が可能であり、そのことで自分自身に対する満足度はある一定のレベルまで高くなったと考えられた。先行報告では Total CIQ は QOLIBRI の Daily life and autonomy と正の相関があった[25]。今回の結果からも有意ではないが正の関係は認められたことから、社会参加

ができることは日常生活を実施する自分の能力に対する満足感と関係がある可能性は否定できない。一方で、Burleigh et. al.は Total CIQ と人生への満足度に直線的な相関はなかったと報告した[48]。本研究においても、TBI 者の社会参加と QOL の関係は直線的関係だけで説明できないことが示唆された。Total CIQ15 点までは QOLIBRI の self は向上を認め、Total CIQ 15 点以降は QOLIBRI の Self はほぼ変化がなかった。CIQ15 点は、CIQ 得点の平均範囲内で自分自身への満足度の向上に関わる上限の指標であると考えられた。このことから、TBI 者の社会参加は一定のレベルまでは QOL に関連するが、それ以上に社会参加を達成しても QOL には関与しないことが示唆され、TBI 者の QOL には社会参加以外の様々な要因（病前の就労[14,15]、教育歴、主観的な自立度[14]、社会的孤立[16]）が関わるという QOL の多面性を反映していることが示唆された。

CIQ-HI においては、Total QOLIBRI とその下位尺度で有意な関連はみられなかった。家事ができることは心身機能や生活自立度が保たれて[25, 36]最小の社会単位である家庭の中で一定の役割を果たすことができることを意味する。しかしながら、家庭内の社会参加では QOL の向上に不十分であることが示唆された。家庭参加について、病前に家庭内での役割が主婦であるかどうか、家事に従事する配偶者の有無により、点数低下する可能性があることを考慮する必要が指摘されており[36]、病前の役割や家族構成を踏まえて、家庭参加に対する支援を行うことである程度までは QOL の向上もできるのではないかと考えられた。

CIQ-SI においては、CIQ-SI8 点まで QOLIBRI の self の向上が認められたことから、社会統合できていることは自分自身に対する満足度と一定のレベルまで関連があることが示唆された。CIQ-SI8 点の点数は平均範囲内であり、社会統合が QOL 向上に関わる上限値の指標と考えられた。このことから、TBI 者の社会統合は一定のレベルまでは QOL に関連するが、それ以上に社会統合を達成したとしても QOL は向上せず、社会統合以外の要因が関わっていることが示唆された。TBI において、社会的孤立と QOL 低下の関連が報告されている[16]。また、CIQ の社会統合と人生への満足度が関連することが報告されている[48]。本研究の対象者は ADL が比較的自立しており、余暇活動をしているのは 6 割程度であったが、外出頻度や家族以外の人と会う頻度が週に数回は保たれていた。TBI は、精神疲労、運動能力の低下、記憶障害、実行機能障害の存在が、買い物、公共交通機関の利用の困難と関連することが報告されている[49]。今回は、高次脳機能障害があり、レジャーや買い物に行くような社会参加は困難な対象が多かったが、外出し家族以外の人と接する機会をもつことが自分自身への満足感を高めることに寄与したと考えられた。このことから、高次脳機能障害があっても、コミュニティへの参加ができるような支援や友人や知人と交流を行うことで、QOL の向上ができるのではないかと考えられた。社交活動や余暇活動への参加推進活動に参加した人は社会参加が高く[50]、受傷後平均 7.7 年経った後天性脳損傷者に対して、社会性とレクリエーションのプログラムを行うことで、社会参加、満足度、家族の介護負担の改善がみられたこと[51]が報告されている。そのため、CIQ-SI が 0-8 点内に該当する TBI 者に対して、他者との交流や余暇活動を促すような支援を行い、社会統合を向上させ、QOL の向上につなぐことが重要であると考える。

CIQ-PS においては、Total QOLIBRI と下位尺度で有意な関連はみられなかった。先行研究においては、生産活動への参加が TBI 者の受傷 20 年後の身体的健康や精神的健康に影響し、病前の就

労[14,15,22]、高い教育歴、主観的な自立度の高さ[14]、仕事への参加が高い QOL と関連する[52]ことが報告されている。

一方で本研究においては、社会人口学的変数と参加者の臨床的特徴別の CIQ の点数による分布の結果より、就労している人はより社会参加していたことから、就労は社会参加を促進することが示唆されたが、生産活動は QOL と関連がみられなかった。先行研究では、生産性と QOLIBRI の Daily life and autonomy と相関していたことが報告されているが[25]、就労人数が不明で SI の点数（平均 2 点）は、今回の対象者の方が高かった（平均 6.2）。今回の母集団は半数以上が就労しており先行研究よりも社会統合が高い集団であったことから、就労自体に伴う問題点が QOL に影響している可能性が考えられた。

TBI における生産性は身体的及び精神的健康の QOL 向上に重要[22]と考えられるが、TBI において、フルタイム雇用よりパートタイム雇用のほうが、ニーズが満たされ、社会に統合できていたことが報告されている[53]。長時間就労によるストレスや交友のための時間が少なくなると、就労していても QOL の向上に関連しない可能性が考えられた。非常に長期的な視点から地域社会への統合と労働参加を最適化するためには、職業リハビリテーションと職業支援を提供し[54,55]、環境的要因を考慮すること[56]の必要性が指摘されている。環境的要因の配慮としては、就労しやすくするだけでなく継続して就労できるように、復職先の職場への情報提供、復職後のサポートが重要と思われる。

TBI の長期的な地域統合の予測因子は、受傷時の年齢、認知機能、身体の痛みであることが報告されている[57]。TBI に対する包括的かつ総合的な神経心理学的リハビリテーションは、地域統合の向上、機能的自立、生産性の向上につなげることができるとされる[58]。集中的認知リハビリテーションは社会参加の改善に有用であり、社会参加および認知レベルに対する満足度と関連があると言われている[59]。そのため、家事、レジャー活動、友人との交流、就労など地域統合のより良い転帰のためにとりわけ急性期からリハビリテーションで認知機能に焦点を当てるべきであろう。つまり、TBI 者に対して急性期から認知機能のリハビリテーションを行うことで、より社会参加を促し QOL の向上を期待できると考える。

今回、Total CIQ と SI が QOLIBRI の self と関連が認められた。外出や他者との交流ができることが TBI の自分自身に対する満足度の向上にある程度まで関連することが示唆された。これは、先行研究[25]で示された直線的に変化する関係ではなく、非線形回帰分析によって QOL の変化を曲線的に捉えることが可能であり、社会参加が QOL に与える影響についてより正確に明らかにすることができたと考える。社会参加の QOL に及ぼす影響に限界があること、QOL には社会参加のみでは促進されない側面が存在することが示唆された。そのため、TBI 者の環境的要因や病前の社会背景、個人因子を考慮した包括的なリハビリテーションや支援がより望まれる。TBI 者が社会的に受け入れられることで孤独が回避され、生産活動の支援体制や私生活を安定させることでさらなる QOL の改善が期待できると考えられた。

研究の限界

本研究の限界としては、受傷前の家庭における具体的役割、就労に伴う疲労やストレスの内容、

また、脳損傷部位の影響を含めた分析をできなかったことがあげられる。

今後の展望として、実際にリハビリや地域あるいは医療による支援で社会参加に変化がみられた TBI 者に対して、HRQOL に改善が認められるか調査していくことがあげられる。

謝辞

本論文投稿に当たり、ご指導いただいた山田晃司先生、鈴木めぐみ先生に謝意を表します。また、統計分析にあたり、ご指導いただいた岐阜大学医学部附属病院 先端医療・臨床研究推進センターの石原拓磨先生に謝意を表します。

利益相反

本論文に関して，開示すべき利益相反関連事項はない。

引用文献　Uncategorized References

1. Goverover Y, Genova H, Smith A, Chiaravalloti N, Lengenfelder J. Changes in activity participation following traumatic brain injury. Neuropsychol Rehabil 2017; 27: 472-85.
2. Hart T, Rabinowitz A. Changes in social participation between 1 and 2 years following moderate-severe traumatic brain injury. Front Rehabil Sci 2022; 3: 945699.
3. Bherer L, Dufour P, Montambeault F. What Is Informal Participation? International Journal of Politics, Culture, and Society 2023; 36: 1-16.
4. Douglas H, Georgiou A, Westbrook J. Social participation as an indicator of successful aging: an overview of concepts and their associations with health. Aust Health Rev 2017; 41: 455-62.
5. Willemse-van Son AH, Ribbers GM, Hop WC, Stam HJ. Community integration following moderate to severe traumatic brain injury: a longitudinal investigation. J Rehabil Med 2009; 41: 521-7.
6. Larsson J, Bjorkdahl A, Esbjornsson E, Sunnerhagen KS. Factors affecting participation after traumatic brain injury. J Rehabil Med 2013; 45: 765-70.
7. Malone C, Erler KS, Giacino JT, Hammond FM, Juengst SB, Locascio JJ, Nakase-Richardson R, Verduzco-Gutierrez M, Whyte J, Zasler N, Bodien YG. Participation Following Inpatient Rehabilitation for Traumatic Disorders of Consciousness: A TBI Model Systems Study. Front Neurol 2019; 10: 1314.
8. Andelic N, Arango-Lasprilla JC, Perrin PB, Sigurdardottir S, Lu J, Landa LO, Forslund MV, Roe C. Modeling of Community Integration Trajectories in the First Five Years after Traumatic Brain Injury. J Neurotrauma 2016; 33: 95-100.
9. Forslund MV, Roe C, Sigurdardottir S, Andelic N. Predicting health-related quality of life 2 years after moderate-to-severe traumatic brain injury. Acta Neurol Scand 2013; 128: 220-7.
10. Chua KS, Kong KH. Rehabilitation outcome following traumatic brain injury--the Singapore experience. Int J Rehabil Res 1999; 22: 189-97.
11. Polinder S, Haagsma JA, van Klaveren D, Steyerberg EW, van Beeck EF. Health-related quality of life after TBI: a systematic review of study design, instruments, measurement properties, and outcome. Popul Health Metr 2015; 13: 4.
12. Guyatt GH, Feeny DH, Patrick DL. Measuring health-related quality of life. Ann Intern Med 1993; 118: 622-9.
13. Black N. Patient reported outcome measures could help transform healthcare. Bmj 2013; 346: f167.
14. Truelle JL, Koskinen S, Hawthorne G et al. Quality of life after traumatic brain injury: the clinical use of the QOLIBRI, a novel disease-specific instrument. Brain Inj 2010; 24: 1272-91.
15. Soberg HL, Røe C, Anke A, Arango-Lasprilla JC, Skandsen T, Sveen U, von Steinbüchel N, Andelic N. Health-related quality of life 12 months after severe traumatic brain injury: a prospective nationwide cohort study. J Rehabil Med 2013; 45: 785-91.

16. Hawthorne G, Kaye AH, Gruen R, Houseman D, Bauer I. Traumatic brain injury and quality of life: initial Australian validation of the QOLIBRI. J Clin Neurosci 2011; 18: 197-202.

17. Berger E, Leven F, Pirente N, Bouillon B, Neugebauer E. Quality of Life after traumatic brain injury: A systematic review of the literature. Restor Neurol Neurosci 1999; 14: 93-102.

18. Sashika H, Takada K, Kikuchi N. Rehabilitation needs and participation restriction in patients with cognitive disorder in the chronic phase of traumatic brain injury. Medicine (Baltimore) 2017; 96: e5968.

19. Matérne M, Strandberg T, Lundqvist LO. Change in quality of life in relation to returning to work after acquired brain injury: a population-based register study. Brain Inj 2018; 32: 1731-9.

20. van Markus-Doornbosch F, van der Holst M, de Kloet AJ, Vliet Vlieland TPM, Meesters JJL. Fatigue, Participation and Quality of Life in Adolescents and Young Adults with Acquired Brain Injury in an Outpatient Rehabilitation Cohort. Dev Neurorehabil 2020; 23: 328-35.

21. Corrigan JD, Bogner J. Latent factors in measures of rehabilitation outcomes after traumatic brain injury. J Head Trauma Rehabil 2004; 19: 445-58.

22. Andelic N, Howe EI, Hellstrom T, Sanchez MF, Lu J, Lovstad M, Roe C. Disability and quality of life 20 years after traumatic brain injury. Brain Behav 2018; 8: e01018.

23. Willer B, Rosenthal M, Kreutzer JS, Gordon WA, Rempel R. Assessment of community integration following rehabilitation for traumatic brain injury. Journal of Head Trauma Rehabilitation 1993; 8: 75-87.

24. World Health O. International classification of impairments, disabilities, and handicaps. Geneva: World Health Organization; 1980.

25. Gerber GJ, Gargaro J, McMackin S. Community integration and health-related quality-of-life following acquired brain injury for persons living at home. Brain Inj 2016; 30: 1552-60.

26. Born K, Amsler F, Gross T. Prospective evaluation of the Quality of Life after Brain Injury (QOLIBRI) score: minor differences in patients with major versus no or mild traumatic brain injury at one-year follow up. Health Qual Life Outcomes 2018; 16: 136.

27. Gorbunova A, Zeldovich M, Voormolen DC, Krenz U, Polinder S, Haagsma JA, Hagmayer Y, Covic A, Real RGL, Asendorf T, von Steinbuchel NV. Reference Values of the QOLIBRI from General Population Samples in the United Kingdom and The Netherlands. J Clin Med 2020; 9.

28. Helmrich I, van Klaveren D, Dijkland SA, Lingsma HF, Polinder S, Wilson L, von Steinbuechel N, van der Naalt J, Maas AIR, Steyerberg EW, Collaborators C-T. Development of prognostic models for Health-Related Quality of Life following traumatic brain injury. Qual Life Res 2022; 31: 451-71.

29. Patel MB, Wilson LD, Bregman JA, Leath TC, Humble SS, Davidson MA, de Riesthal MR, Guillamondegui OD. Neurologic Functional and Quality of Life Outcomes after TBI: Clinic Attendees versus Non-Attendees. J Neurotrauma 2015; 32: 984-9.

30. Rasmussen MS, Andelic N, Pripp AH, Nordenmark TH, Soberg HL. The effectiveness of a

family-centred intervention after traumatic brain injury: A pragmatic randomised controlled trial. Clin Rehabil 2021; 35: 1428-41.

31. Siponkoski ST, Wilson L, von Steinbuchel N, Sarajuuri J, Koskinen S. Quality of life after traumatic brain injury: Finnish experience of the QOLIBRI in residential rehabilitation. J Rehabil Med 2013; 45: 835-42.

32. von Steinbüchel N, Real RGL, Sasse N et al. German validation of Quality of Life after Brain Injury (QOLIBRI) assessment and associated factors. PLoS One 2017; 12: e0176668.

33. von Steinbuchel N, Wilson L, Gibbons H et al. Quality of Life after Brain Injury (QOLIBRI): scale validity and correlates of quality of life. J Neurotrauma 2010; 27: 1157-65.

34. von Steinbuechel N, Covic A, Polinder S et al. Assessment of Health-Related Quality of Life after TBI: Comparison of a Disease-Specific (QOLIBRI) with a Generic (SF-36) Instrument. Behav Neurol 2016; 2016: 7928014.

35. Wilson L, Marsden-Loftus I, Koskinen S, Bakx W, Bullinger M, Formisano R, Maas A, Neugebauer E, Powell J, Sarajuuri J, Sasse N, von Steinbuechel N, von Wild K, Truelle JL. Interpreting Quality of Life after Brain Injury Scores: Cross-Walk with the Short Form-36. J Neurotrauma 2017; 34: 59-65.

36. Sander AM, Fuchs KL, High WM, Jr., Hall KM, Kreutzer JS, Rosenthal M. The Community Integration Questionnaire revisited: an assessment of factor structure and validity. Arch Phys Med Rehabil 1999; 80: 1303-8.

37. Teasdale G, Jennett B. Assessment of coma and impaired consciousness. A practical scale. Lancet 1974; 2: 81-4.

38. Wilson JT, Pettigrew LE, Teasdale GM. Structured interviews for the Glasgow Outcome Scale and the extended Glasgow Outcome Scale: guidelines for their use. J Neurotrauma 1998; 15: 573-85.

39. Zigmond AS, Snaith RP. The hospital anxiety and depression scale. Acta Psychiatr Scand 1983; 67: 361-70.

40. Snaith RP. The Hospital Anxiety And Depression Scale. Health and Quality of Life Outcomes 2003; 1: 29.

41. von Steinbuechel N, Petersen C, Bullinger M, Group Q. Assessment of health-related quality of life in persons after traumatic brain injury--development of the Qolibri, a specific measure. Acta Neurochir Suppl 2005; 93: 43-9.

42. Suzuki M, Naito M, Ota K, von Wild KRH, Saitoh E, Kondo I. Validation of the Japanese Version of the Quality of Life after Brain Injury (QOLIBRI) Scale. Brain & Neurorehabilitation 2019; 12.

43. Mollayeva T, Shapiro CM, Mollayeva S, Cassidy JD, Colantonio A. Modeling community integration in workers with delayed recovery from mild traumatic brain injury. BMC Neurol 2015; 15: 194.

44. Saeki S, Okazaki T, Hachisuka K. Concurrent validity of the Community Integration

Questionnaire in patients with traumatic brain injury in Japan. J Rehabil Med 2006; 38: 333-5.
45. Schmidt MF, Garvin LJ, Heinemann AW, Kelly JP. Gender- and age-related role changes following brain injury. The Journal of Head Trauma Rehabilitation 1995; 10: 14-27.
46. Stalnacke BM. Community integration, social support and life satisfaction in relation to symptoms 3 years after mild traumatic brain injury. Brain Inj 2007; 21: 933-42.
47. Williams G, Willmott C. Higher levels of mobility are associated with greater societal participation and better quality-of-life. Brain Inj 2012; 26: 1065-71.
48. Burleigh SA, Farber RS, Gillard M. Community integration and life satisfaction after traumatic brain injury: long-term findings. Am J Occup Ther 1998; 52: 45-52.
49. Mazaux JM, Masson F, Levin HS, Alaoui P, Maurette P, Barat M. Long-term neuropsychological outcome and loss of social autonomy after traumatic brain injury. Arch Phys Med Rehabil 1997; 78: 1316-20.
50. McLean AM, Jarus T, Hubley AM, Jongbloed L. Differences in social participation between individuals who do and do not attend brain injury drop-in centres: a preliminary study. Brain Inj 2012; 26: 83-94.
51. Gerber GJ, Gargaro J. Participation in a social and recreational day programme increases community integration and reduces family burden of persons with acquired brain injury. Brain Inj 2015; 29: 722-9.
52. Steadman-Pare D, Colantonio A, Ratcliff G, Chase S, Vernich L. Factors associated with perceived quality of life many years after traumatic brain injury. J Head Trauma Rehabil 2001; 16: 330-42.
53. O'Neill J, Hibbard MR, Brown M, Jaffe M, Sliwinski M, Vandergoot D, Weiss MJ. The effect of employment on quality of life and community integration after traumatic brain injury. J Head Trauma Rehabil 1998; 13: 68-79.
54. Brown AW, Moessner AM, Mandrekar J, Diehl NN, Leibson CL, Malec JF. A survey of very-long-term outcomes after traumatic brain injury among members of a population-based incident cohort. J Neurotrauma 2011; 28: 167-76.
55. Ditchman N, Thomas JA, Johnson K, Haak C, Rafajko S. The impact of employment on quality of life for adults with brain injury. Journal of Vocational Rehabilitation 2022; 56: 1-15.
56. Forslund MV, Roe C, Arango-Lasprilla JC, Sigurdardottir S, Andelic N. Impact of personal and environmental factors on employment outcome two years after moderate-to-severe traumatic brain injury. J Rehabil Med 2013; 45: 801-7.
57. Lu J, Rasmussen MS, Sigurdardottir S, Forslund MV, Howe EI, Fure SCR, Lovstad M, Overeem R, Roe C, Andelic N. Community Integration and Associated Factors 10 Years after Moderate-to-Severe Traumatic Brain Injury. J Clin Med 2023; 12.
58. Cicerone KD, Langenbahn DM, Braden C, Malec JF, Kalmar K, Fraas M, Felicetti T, Laatsch L, Harley JP, Bergquist T, Azulay J, Cantor J, Ashman T. Evidence-based cognitive rehabilitation:

updated review of the literature from 2003 through 2008. Arch Phys Med Rehabil 2011; 92: 519-30.

59. Cicerone KD, Mott T, Azulay J, Friel JC. Community integration and satisfaction with functioning after intensive cognitive rehabilitation for traumatic brain injury. Arch Phys Med Rehabil 2004; 85: 943-50.

図のキャプション

Figure 1　Total CIQ と Total QOLIBRI の関連
非線形回帰分析 (p=0.154)
Total CIQ：Community Integration Questionnaire 総得点
Total QOLIBRI：Quality of Life after Brain Injury 総得点

Figure 2　HI と Total QOLIBRI の関連
非線形回帰分析(p=0.859)
HI：Home integration scale
Total QOLIBRI：Quality of Life after Brain Injury 総得点

Figure 3　SI と Total QOLIBRI の関連
非線形回帰分析(p=0.362)
SI：Social integration scale
Total QOLIBRI：Quality of Life after Brain Injury 総得点

Figure 4　PS と QOLIBRI の関連
非線形回帰分析(p=0.084)
PS：Productivity scale
Total QOLIBRI：Quality of Life after Brain Injury 総得点

Figure 5　Total CIQ と Self の関連
非線形回帰分析(p=0.006)
Total CIQ：Community Integration Questionnaire 総得点

Figure 6　SI と Self の関連
非線形回帰分析(p=0.018)
SI：Social integration scale

図

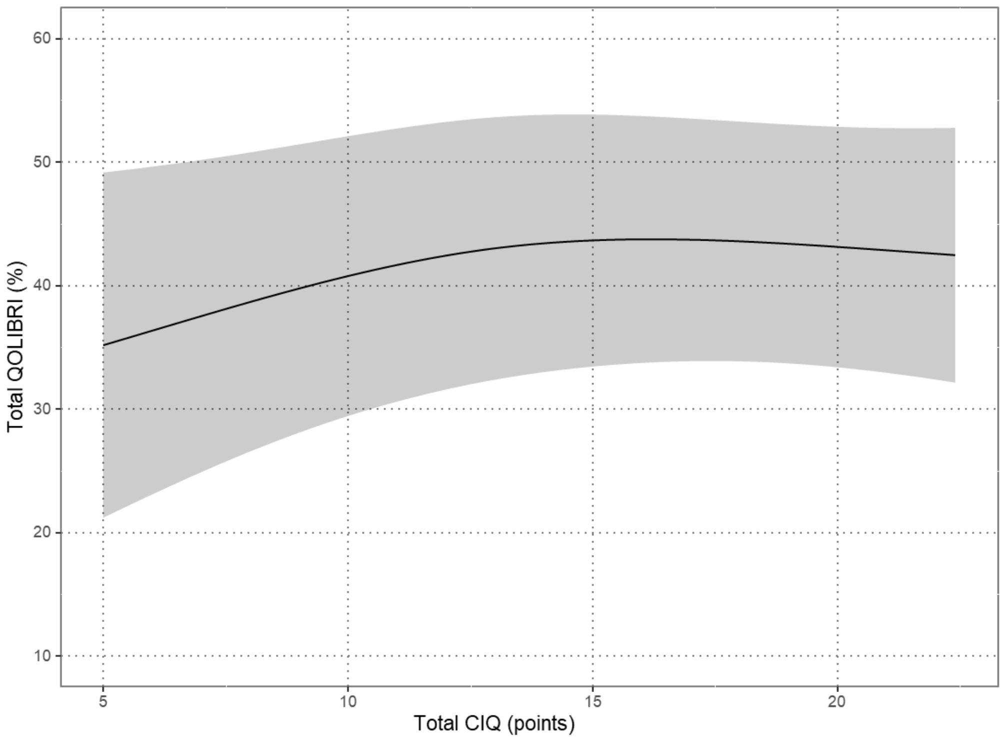

Figure1.
縦軸は QOLIBRI 下位尺度のスコア（%）、横軸は Total CIQ (points)のスコアを示す。

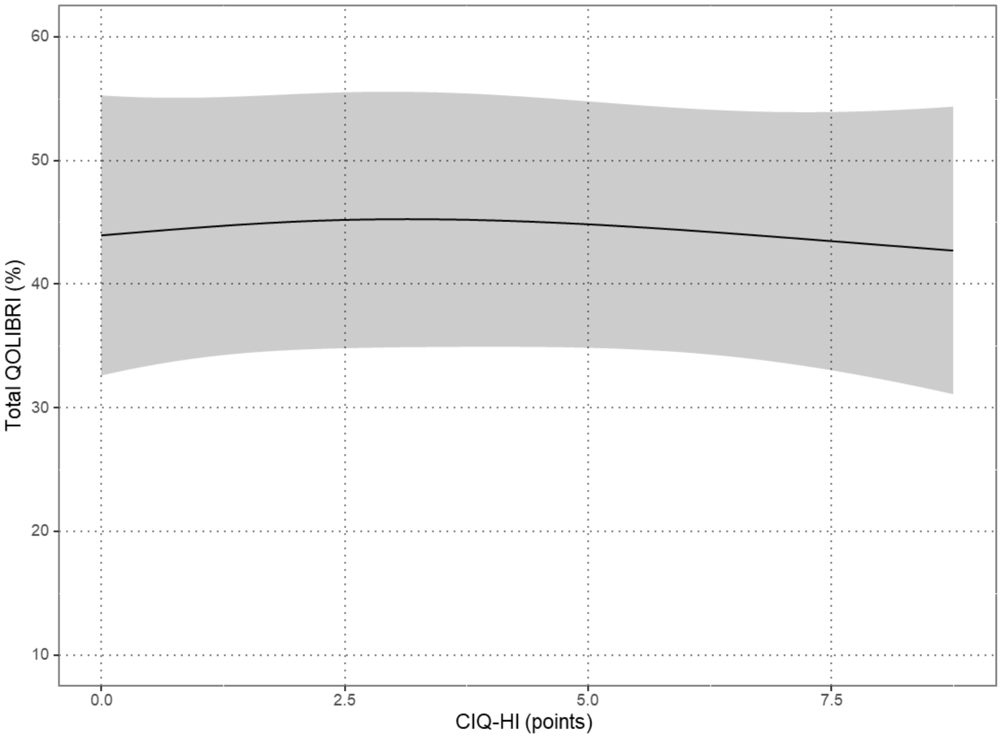

Figure 2.

縦軸は QOLIBRI 下位尺度のスコア（%）、横軸は CIQ-HI (points)のスコアを示す。

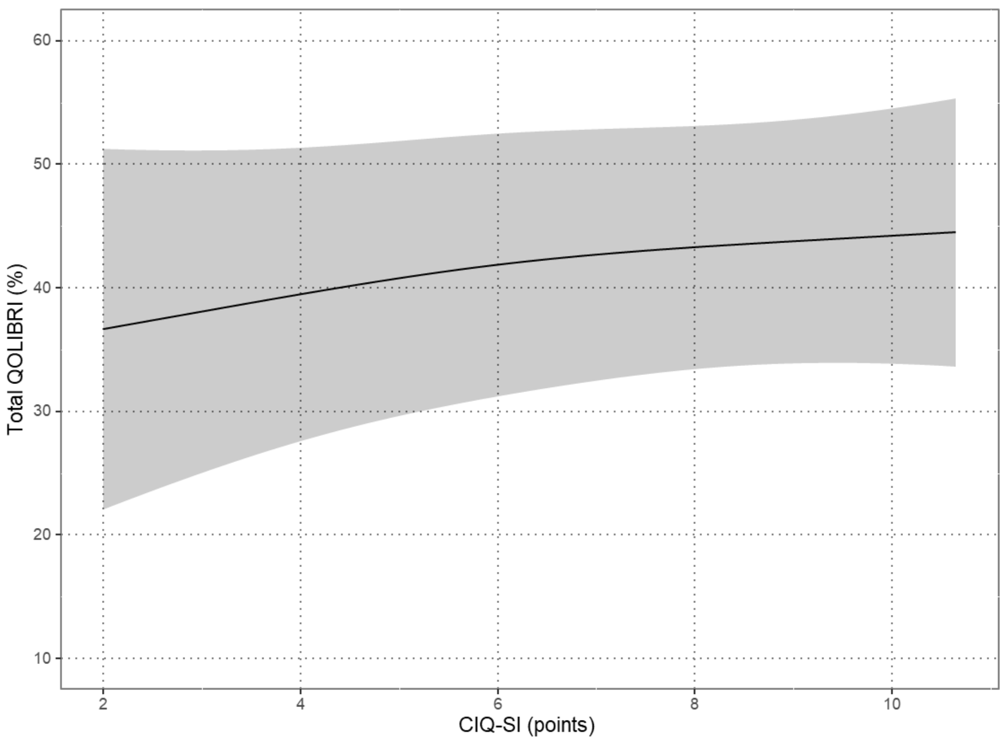

Figure 3.
縦軸は QOLIBRI 下位尺度のスコア（%）、横軸は CIQ-SI (points)のスコアを示す。

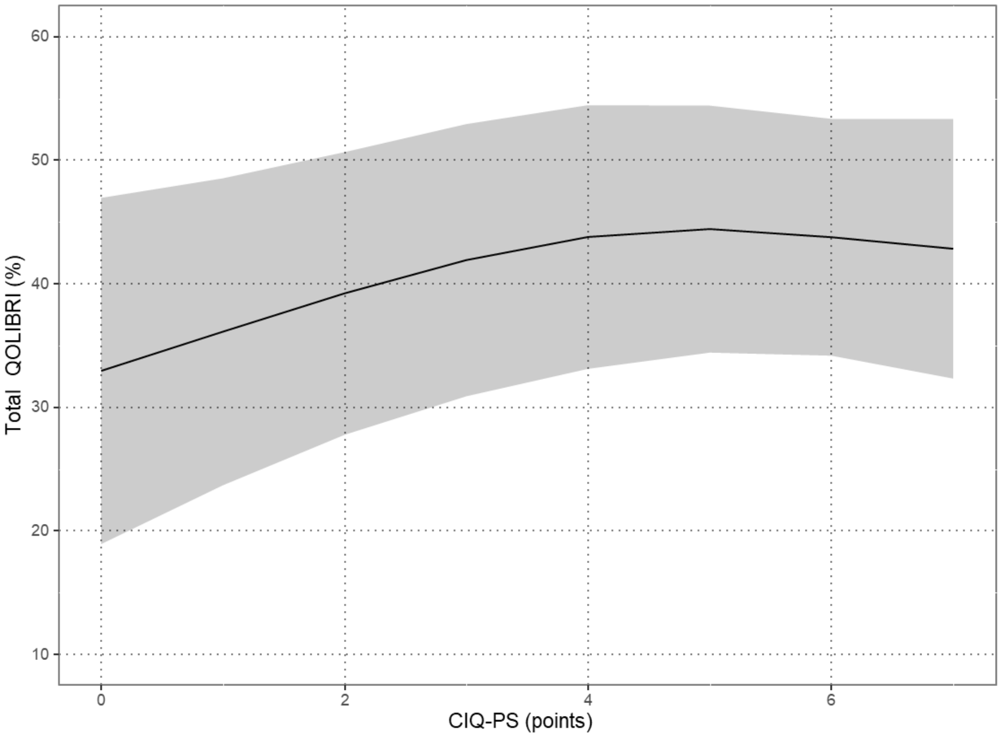

Figure 4.
縦軸は QOLIBRI 下位尺度のスコア（%）、横軸は CIQ-PS (points)のスコアを示す。

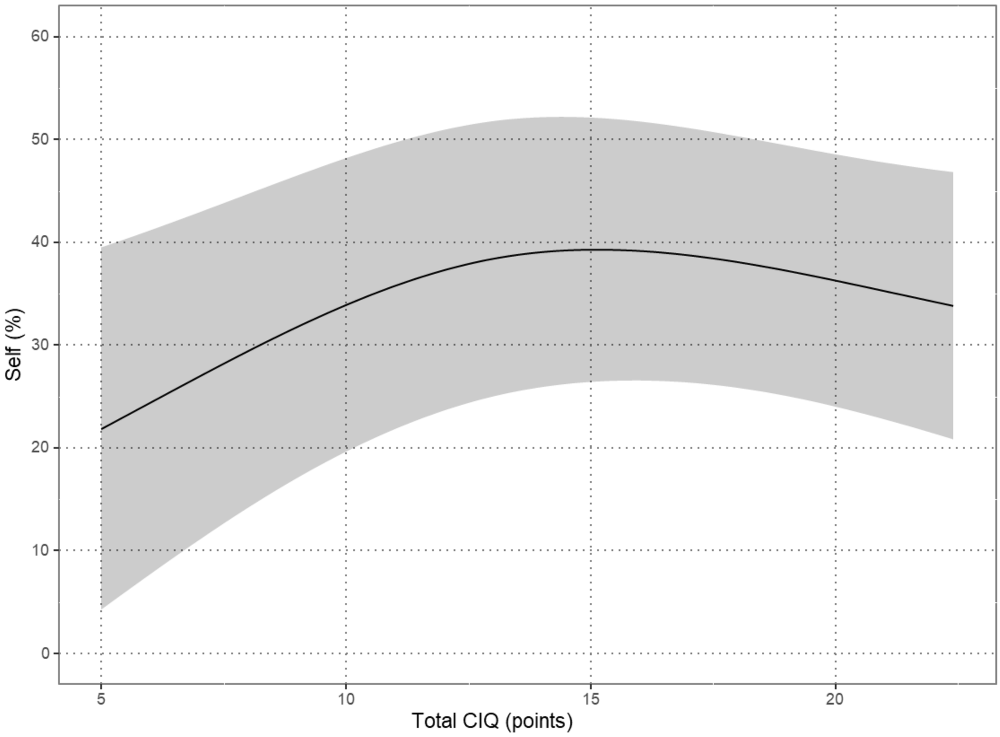

Figure 5.
縦軸は QOLIBRI 下位尺度 self のスコア（%）、横軸は Total CIQ (points)のスコアを示す。

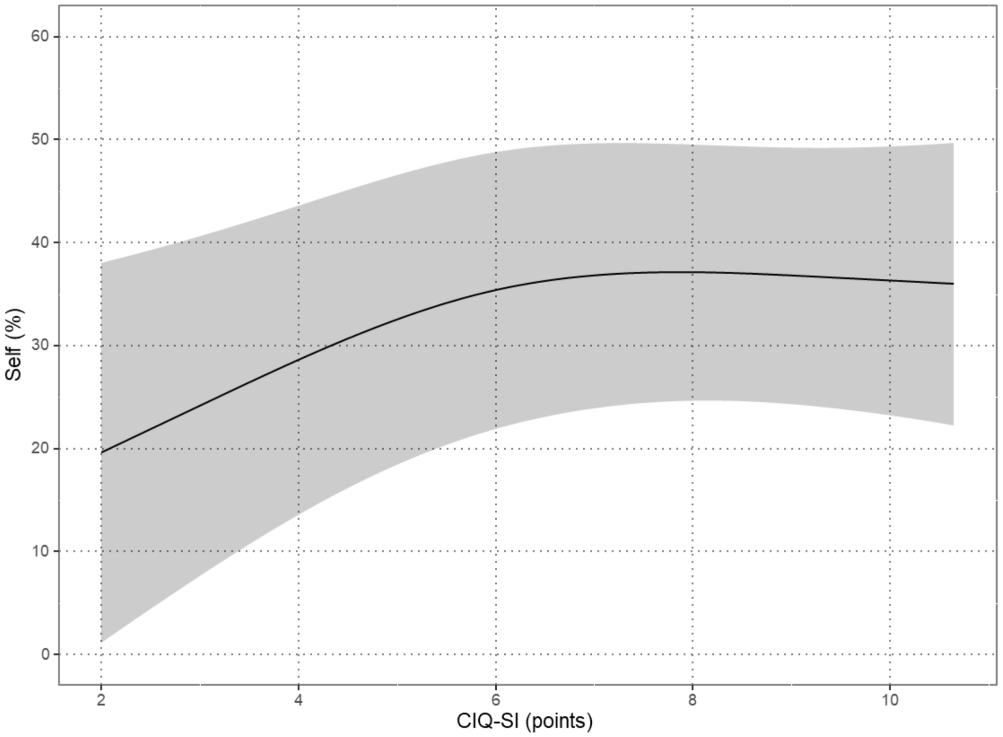

Figure 6.
縦軸は QOLIBRI 下位尺度 self のスコア（%）、横軸は CIQ-SI のスコアを示す。

表

Table1-1. CIQ の点数別の社会人口統計学的特性

Variable	CIQ score 0~9 (n=31)	10~19 (n=75)	20~29 (n=22)	p-value
Age (years)	41 (36, 60)	40 (31, 52)	38 (27, 46)	0.182
Sex (people)				0.424
Female	4 (12.9%)	15 (20.0%)	6 (27.3%)	
Male	27 (87.1%)	60 (80.0%)	16 (72.7%)	
Marital Status (people)				0.13
with partner	14 (45.2%)	34 (45.3%)	8 (36.4%)	
separated from partner	1 (3.2%)	5 (6.7%)	5 (22.7%)	
Single	16 (51.6%)	36 (48.0%)	9 (40.9%)	
education years				0.591
9	5(15.2%)	8(10.7%)	3(15.0%)	
12	18(54.5%)	28(37.3%)	8(40.0%)	
15-16	9(27.3%)	36(48.0%)	8(40.0%)	
others	1(3.0%)	3(4.0%)	1(3.9%)	
Employment Status (people)				**<0.001**
Employed	9 (30.0%)	45 (61.6%)	18 (81.8%)	
Not employed	21 (70.0%)	28 (38.4%)	4 (18.2%)	

*Bold means p-value p<0.05.　Pearson’s chi-square test、Kruskal-Wallis test

年齢の括弧は、CIQ の点数の 3 群（0-9 点、10-19 点、20-29 点）において、変数の四分位範囲の 25%と 75%を示す。

CIQ: Community Integration Questionnaire

Table1-2. CIQ の点数別の社会人口統計学的特性および臨床的特徴

Variable	CIQ score 0~9 (n=31)	10~19(n=75)	20~29 (n=22)	p-value
Major Lesion of Brain (people)				0.651
diffuse	16 (59.3%)	31 (43.7%)	9 (45.0%)	
none on image	0 (0.0%)	7 (9.9%)	2 (10.0%)	
occipital lobe	2 (7.4%)	4 (5.6%)	1 (5.0%)	
frontal lobe	9 (33.3%)	26 (36.6%)	8 (40.0%)	
temporal lobe	0 (0.0%)	3 (4.2%)	0 (0.0%)	
GOSE score (points)	3.0 (3.0, 4.0)	5.0 (4.0, 6.0)	6.0 (5.2, 6.0)	**<0.001**
HADS Anxiety (points)	6.0 (4.0, 10.0)	6.0 (3.0, 9.0)	9.0 (7.0, 10.0)	**0.022**
HADS Depression (points)	12.0 (7.5, 15.0)	7.0 (3.0, 11.0)	8.0 (5.5, 10.8)	**<0.001**
Self-reported independent status (people)				**<0.001**
Completely independent	1 (3.2%)	11 (14.7%)	4 (18.2%)	
Mostly independent	3 (9.7%)	31 (41.3%)	13 (59.1%)	
A little bit independent	12 (38.7%)	27 (36.0%)	4 (18.2%)	
Not independent at all	15 (48.4%)	6 (8.0%)	1 (4.5%)	
Self-reported health status (people)				0.138
Healthy	15 (48.4%)	51 (68.9%)	14 (63.6%)	
Unhealthy	16 (51.6%)	23 (31.1%)	8 (36.4%)	
Rehabilitation (people)				
previously	28 (90.3%)	60 (80.0%)	16 (72.7%)	0.246
Actually	14 (45.2%)	28 (37.3%)	6 (27.3%)	0.415
Clinical Symptoms (people)				
epilepsy	6 (19.4%)	19 (25.3%)	6 (27.3%)	0.755
hemiparesis	12 (38.7%)	18 (24.0%)	2 (9.1%)	**0.047**
visual deficit	11 (35.5%)	10 (13.3%)	1 (4.5%)	**0.005**
auditory deficit	2 (6.5%)	2 (2.7%)	2 (9.1%)	0.395
extra injury	18 (58.1%)	35 (46.7%)	5 (22.7%)	**0.037**
communication disorder	25 (80.6%)	39 (52.0%)	12 (54.5%)	**0.021**
attention disorder	27 (87.1%)	60 (80.0%)	18 (81.8%)	0.687
memory disorder	29 (93.5%)	52 (69.3%)	13 (59.1%)	**0.009**
executive function disorder	29 (93.5%)	50 (66.7%)	14 (63.6%)	**0.011**
affective and behavior disorder	24 (77.4%)	39 (52.0%)	11 (50.0%)	**0.039**

*Bold means p-value p<0.05　Pearson’s chi-square test、Kruskal-Wallis test

GOSE、HADS の括弧は、CIQ の点数の 3 群（0-9 点、10-19 点、20-29 点）において、変数の四分位範囲の 25%と 75%を示す。

CIQ: Community Integration Questionnaire

GOSE: Glasgow Outcome Scale-Extended

HADS: Hospital Anxiety and Depression Scale

Table2-1. Total CIQ と QOLIBRI の関連

Variables	Coefficient	95%LCI	95%UCI	p-value
Total QOLIBRI-Total CIQ	3.231	-1.182	7.645	0.154
Cognition-Total CIQ	3.659	-2.341	9.659	0.444
Self-Total CIQ	5.405	-0.159	10.969	**0.006**
Daily life and autonomy-Total CIQ	5.248	-1.641	12.137	0.18
Social relationships-Total CIQ	4.215	-2.266	10.697	0.409
Emotions-Total CIQ	-2.814	-10.588	4.961	0.713
Physical problems-Total CIQ	1.593	-6.135	9.322	0.56

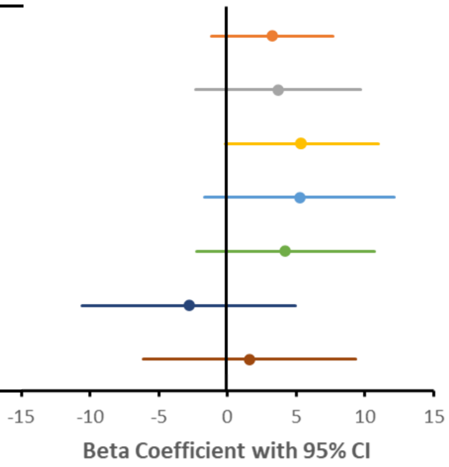

Total CIQ [interquartile range; 25%-75%]= [9.688-17.125]

CIQ：Community Integration Questionnaire

Table2-2. HI と QOLIBRI の関連

Variables	Coefficient	95%LCI	95%UCI	p-value
Total QOLIBRI-CIQ-HI	0.88	-5.944	7.704	0.859
Cognition-CIQ-HI	1.357	-7.823	10.537	0.938
Self-CIQ-HI	5.191	-3.627	14.008	0.296
Daily life and autonomy-CIQ-HI	-0.239	-10.882	10.404	0.933
Social relationships-CIQ-HI	1.186	-8.681	11.053	0.545
Emotions-CIQ-HI	-5.991	-17.689	5.707	0.325
Physical problems-CIQ-HI	2.249	-9.355	13.853	0.21

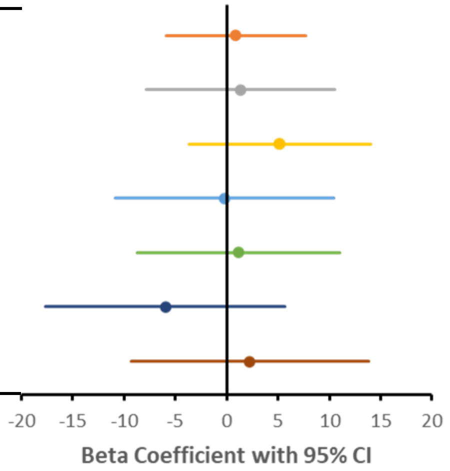

HI [interquartile range; 25%-75%]= [0-5]

HI：Home integration scale

Table2-3. SI と QOLIBRI の関連

Variables	Coefficient	95%LCI	95%UCI	p-value
Total QOLIBRI-CIQ-SI	3.797	-1.489	9.083	0.362
Cognition-CIQ-SI	4.589	-2.529	11.708	0.437
Self-CIQ-SI	8.519	1.832	15.207	**0.018**
Daily life and autonomy-CIQ-SI	4.189	-4.091	12.469	0.589
Social relationships-CIQ-SI	6.353	-1.257	13.964	0.156
Emotions-CIQ-SI	-4.778	-13.982	4.427	0.581
Physical problems-CIQ-SI	0.991	-8.235	10.216	0.968

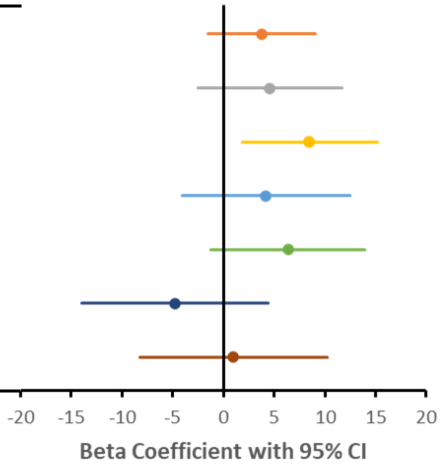

SI[interquartile range; 25%-75%]= [4-8]

SI：Social integration scale

Table2-4. PS と QOLIBRI の関連

Variables	Coefficient	95%LCI	95%UCI	p-value
Total QOLIBRI-CIQ-PS	2.463	-3.338	8.265	0.084
Cognition-CIQ-PS	2.447	-5.532	10.426	0.707
Self-CIQ-PS	-0.15	-7.768	7.468	0.128
Daily life and autonomy-CIQ-PS	5.701	-3.373	14.774	0.119
Social relationships-CIQ-PS	6.328	-2.16	14.815	0.159
Emotions-CIQ-PS	-4.76	-15.023	5.503	0.579
Physical problems-CIQ-PS	4.155	-5.83	14.139	0.062

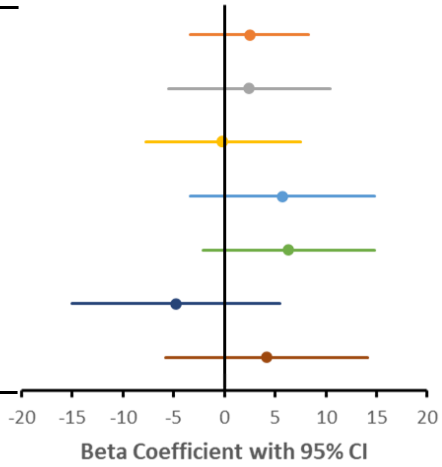

PS[interquartile range; 25%-75%]= [2.75-6]

PS：Productivity scale

非線形回帰分析、p-value の太字は p<0.05 を示す。

CIQ：Community Integration Questionnaire

HI：Home integration scale

SI：Social integration scale

PS：Productivity scale

QOLIBRI：Quality of Life after Brain Injury

CIQ と QORIBRI の非線形回帰分析の Beta 係数を forest plot で示した。色付きの実線は、予測値の 95%信頼区間を示す。実線の中央の点は、Beta 係数を示す。

LCI：Lower confidence interval

UCI：Upper confidence interval

CI：confidence interval

付録

Table.1 CIQ の評価項目と内容

CIQ sub scale	content
Home integration	Shopping for necessities
	Prepares meals
	Housework
	Caring for children
	Social arrangements
Social integration	Personal finances
	Frequency of shopping
	Frequency of leisure activities
	Frequency of visiting friends or relatives
	Who do you participate in leisure activities with?
	Having a best friend
Productivity	Travel outside of home
	Work
	School
	Volunteer work

CIQ: Community Integration Questionnaire

Table.2 QOLIBRI の評価項目と内容

QOLIBRI sub scale	Content
Cognition	Cognitive problems such as memory, attention, expressive speech, and decision making
Self	Aspects of self, including energy, motivation, physical appearance, and self-esteem
Daily Life and autonomy	Independence, activities of daily life, and participation in social roles
Social relationships	Relationships with friends, family, and partner "Bothered" items
Emotions	Feelings of depression, anxiety, loneliness, boredom, and anger
Physical problems	Physical problems, such as slowness, pain, sensory impairment, or other consequences of injury

QOLIBRI: Quality of Life after Brain Injury

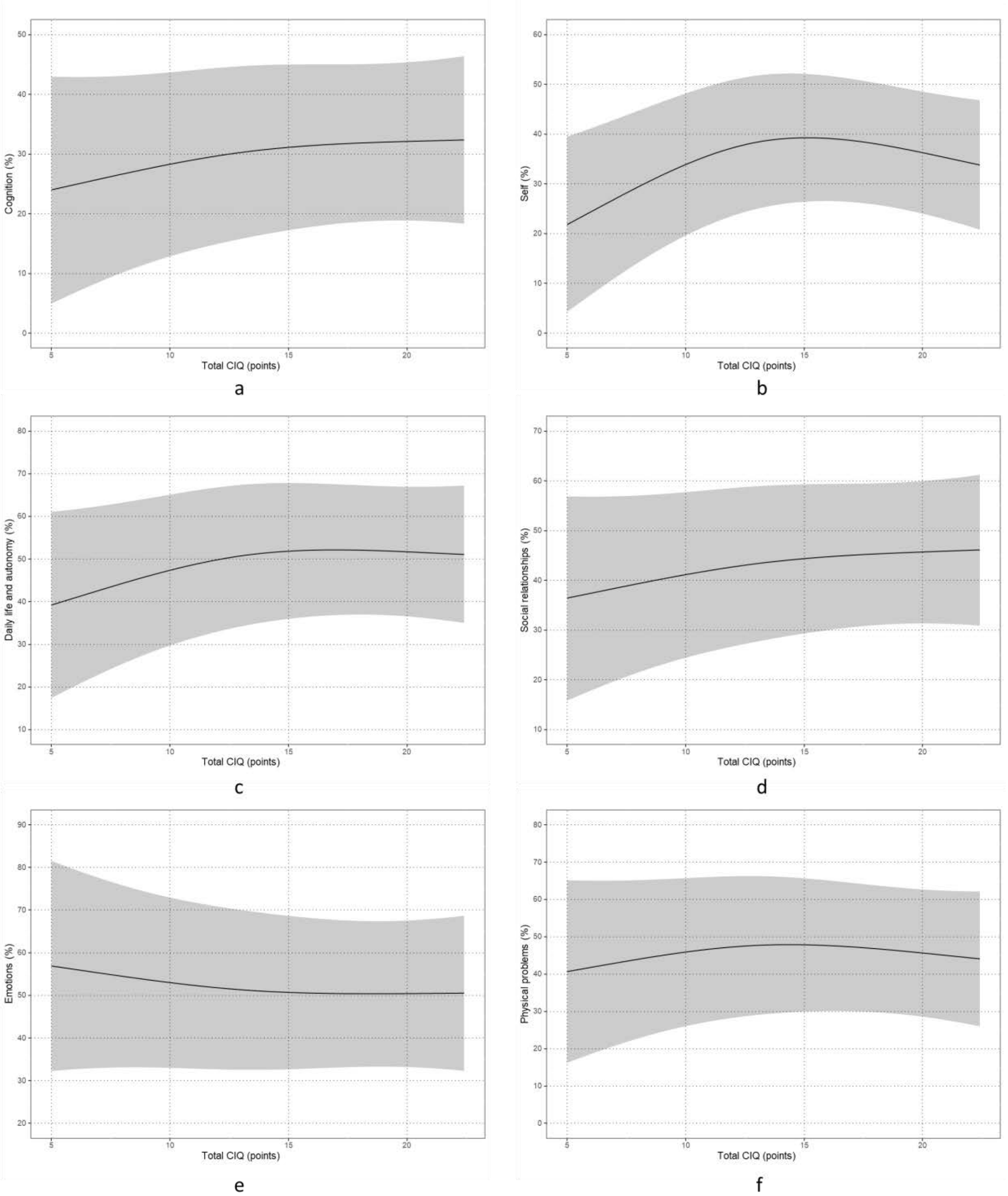

Figure 1. Total CIQ と QOLIBRI 下位尺度の関連

縦軸は QOLIBRI 下位尺度のスコア（%）、横軸は CIQ-HI のスコアを示す。

非線形回帰分析: a) p=0.444　b)p=0.006　c) p=0.18　d) p=0.409　e) p=0.713 f) p=0.56

Total CIQ：Community Integration Questionnaire 総得点

QOLIBRI：Quality of Life after Brain Injury

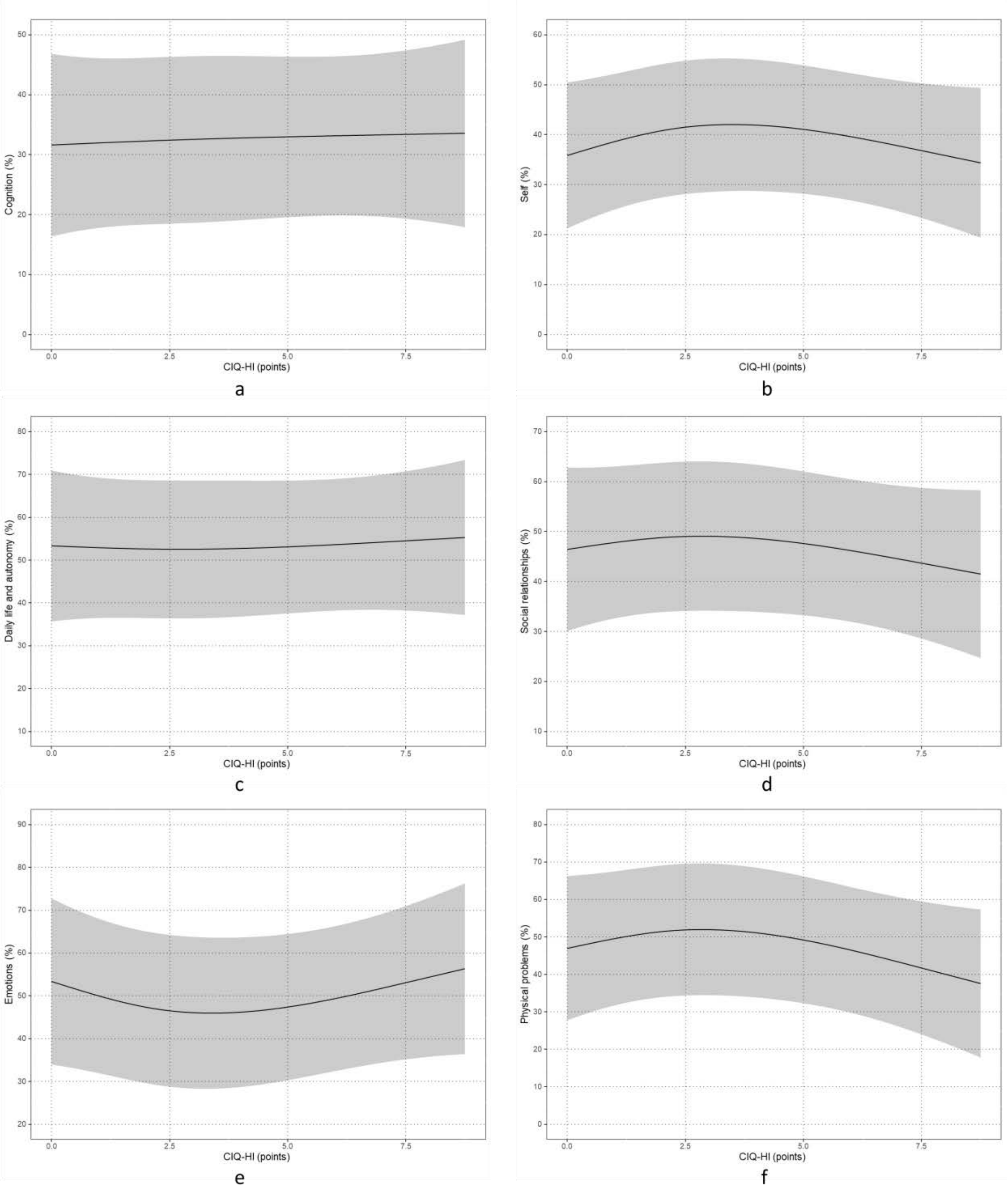

Figure2. CIQ-HI と QOLIBRI 下位尺度の関連

縦軸は QOLIBRI 下位尺度のスコア（%）、横軸は CIQ-HI のスコアを示す。

非線形回帰分析: a) p=0.938　b)p=0.296　c) p=0.933　d) p=0.545　e) p=0.325　f) p=0.21

HI：Home integration scale

QOLIBRI：Quality of Life after Brain Injury

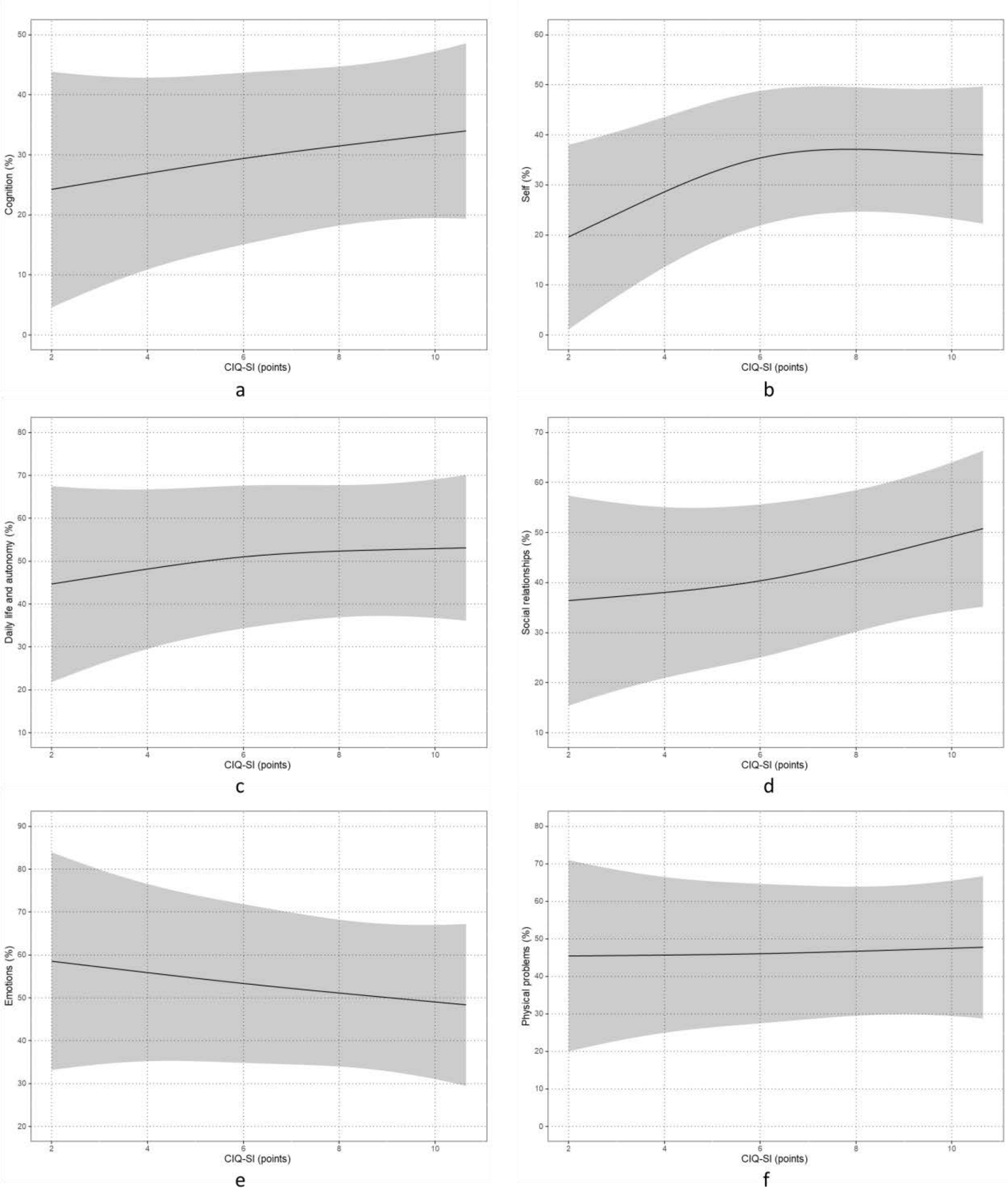

Figure 3. CIQ-SI と QOLIBRI 下位尺度の関連

非線形回帰分析: a) p=0.437　b)p=0.018　c) p=0.589　d) p=0.156　e) p=0.581　f) p=0.968

SI：Social integration scale

QOLIBRI：Quality of Life after Brain Injury

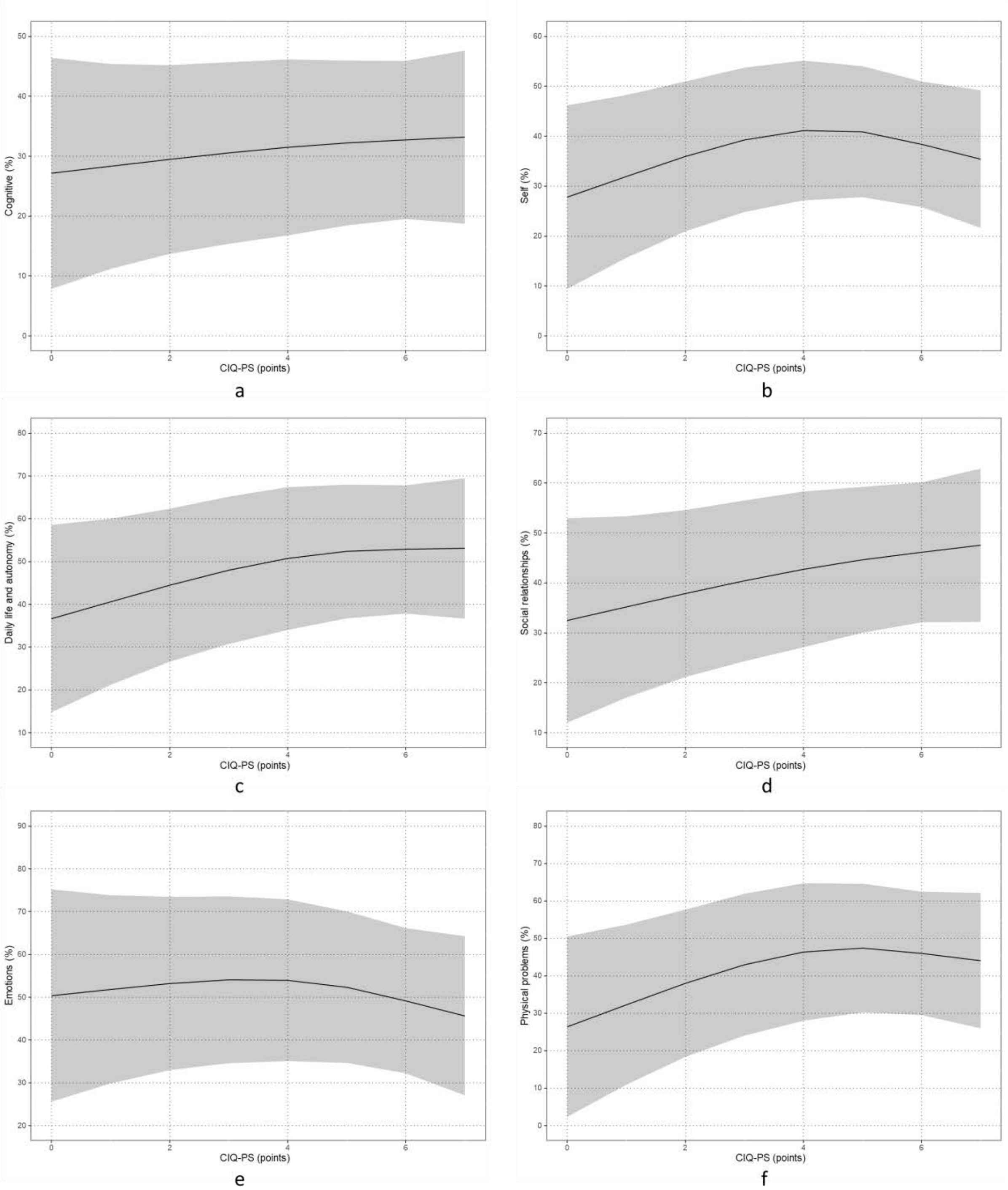

Figure 4. PS と QOLIBRI 下位尺度の関連

非線形回帰分析: a) p=0.707　b)p=0.128　c) p=0.119　d) p=0.159　e) p=0.579　f) p=0.062

PS：Productivity scale

QOLIBRI：Quality of Life after Brain Injury
