# Supplementary material for: The relationship between social participation and quality of life in individuals with traumatic brain injury
Source: Fujita Med J. 2025 Apr 17;11(3):111–20. doi: 10.20407/fmj.2024-016 (PMC12327209; doi:10.20407/fmj.2024-016)
Supplement: Supplementary file 2 — Supplementary Figures [file fmj-11-111-s002.pdf]

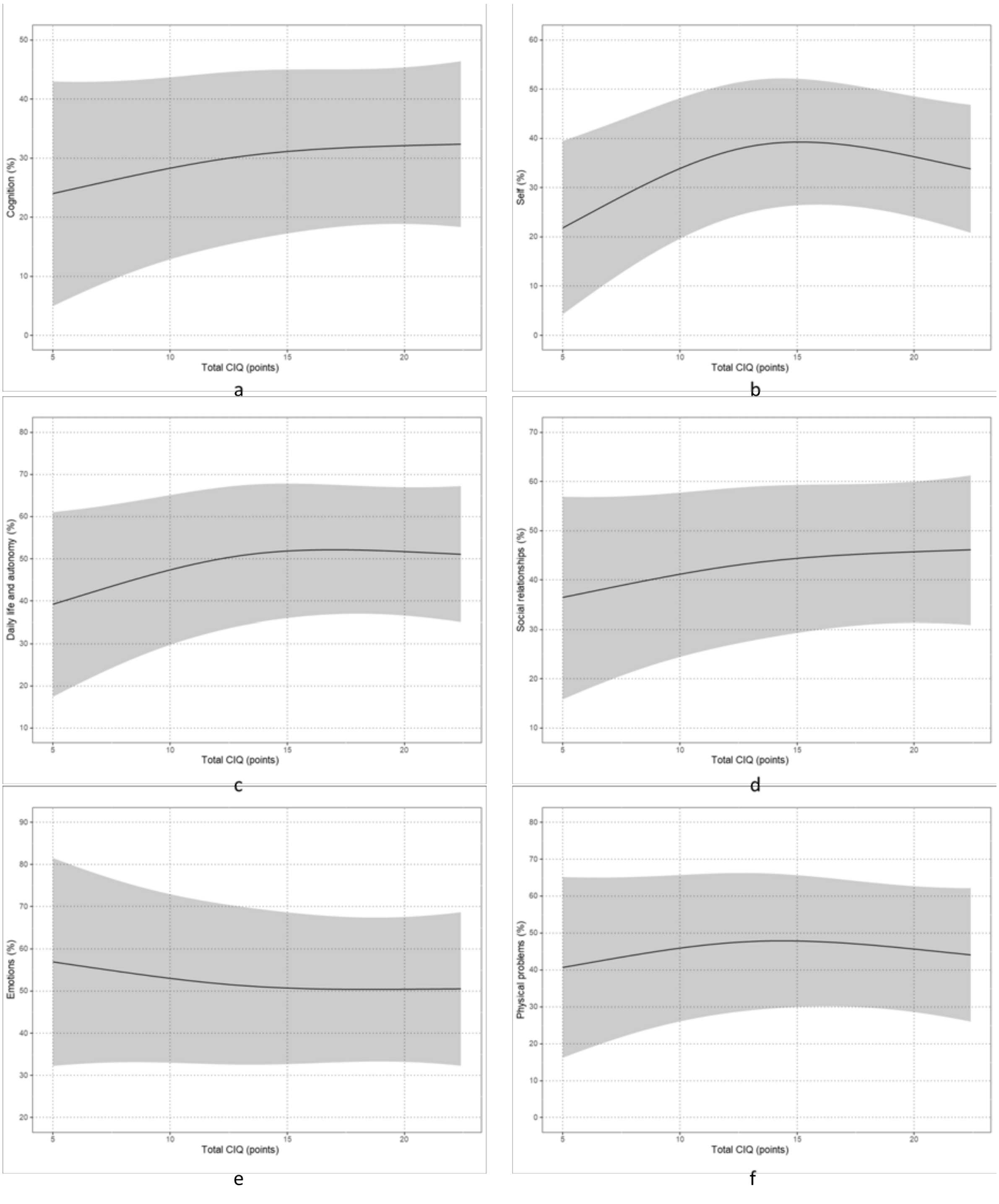

Supplementary figures

Figure 1: Relationship Between Total CIQ and QOLIBRI Subscale

The vertical axis displays the QOLIBRI subscale score (%) and the horizontal axis shows the CIQ-HI score.

Nonlinear Regression Analysis: a) p=0.444 b) p=0.006 c) p=0.18 d) p=0.409 e) p=0.713 f) p=0.56

Total CIQ: Community Integration Questionnaire Total Score

QOLIBRI: Quality of Life after Brain Injury

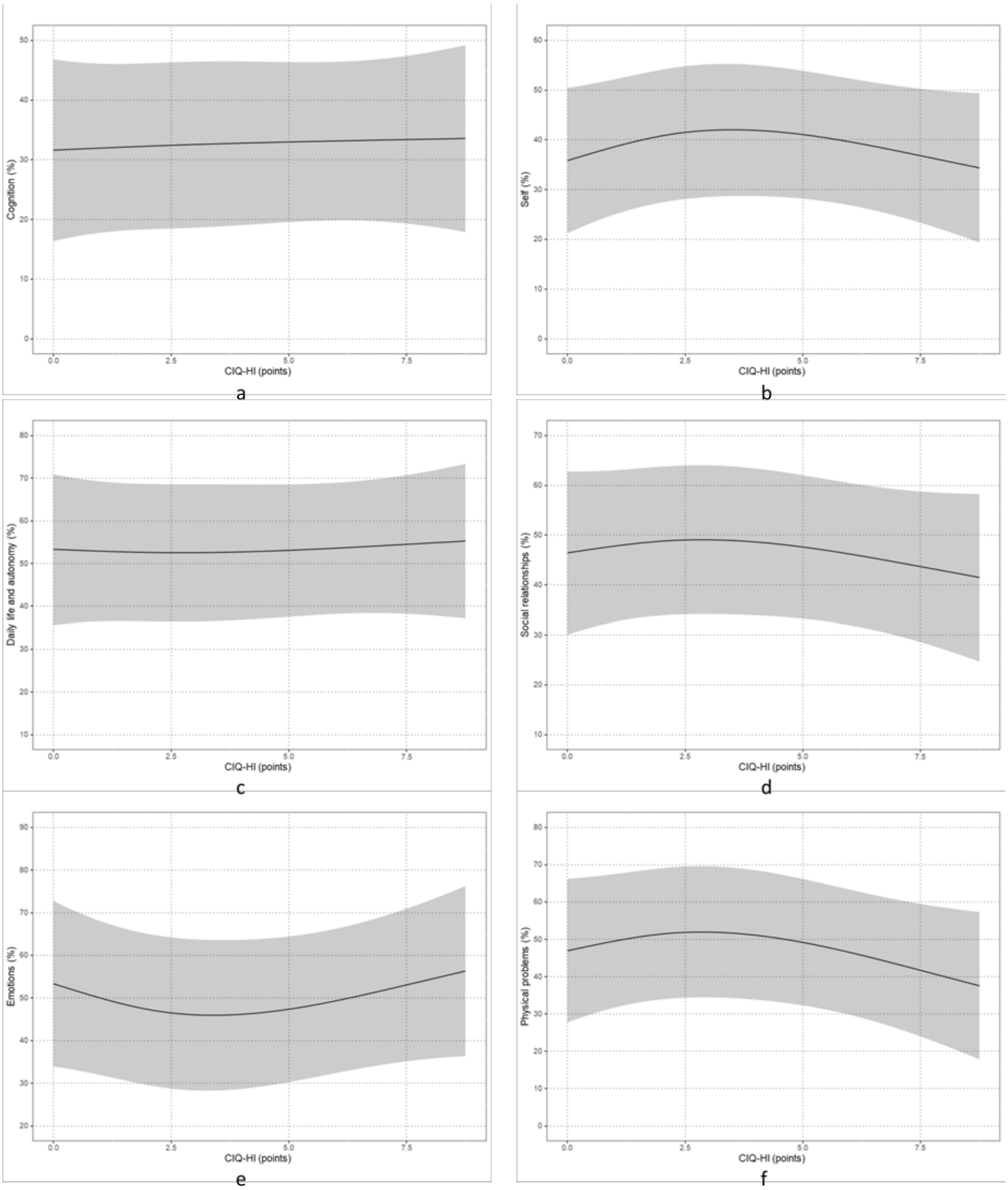

Figure 2: Relationship Between CIQ-HI and QOLIBRI Subscale

The vertical axis displays the QOLIBRI subscale score (%), and the horizontal axis shows the CIQ-HI score.

Nonlinear Regression Analysis: a) p=0.938 b) p=0.296 c) p=0.933 d) p=0.545 e) p=0.325 f) p=0.21

HI: Home Integration Scale

QOLIBRI: Quality of Life after Brain Injury

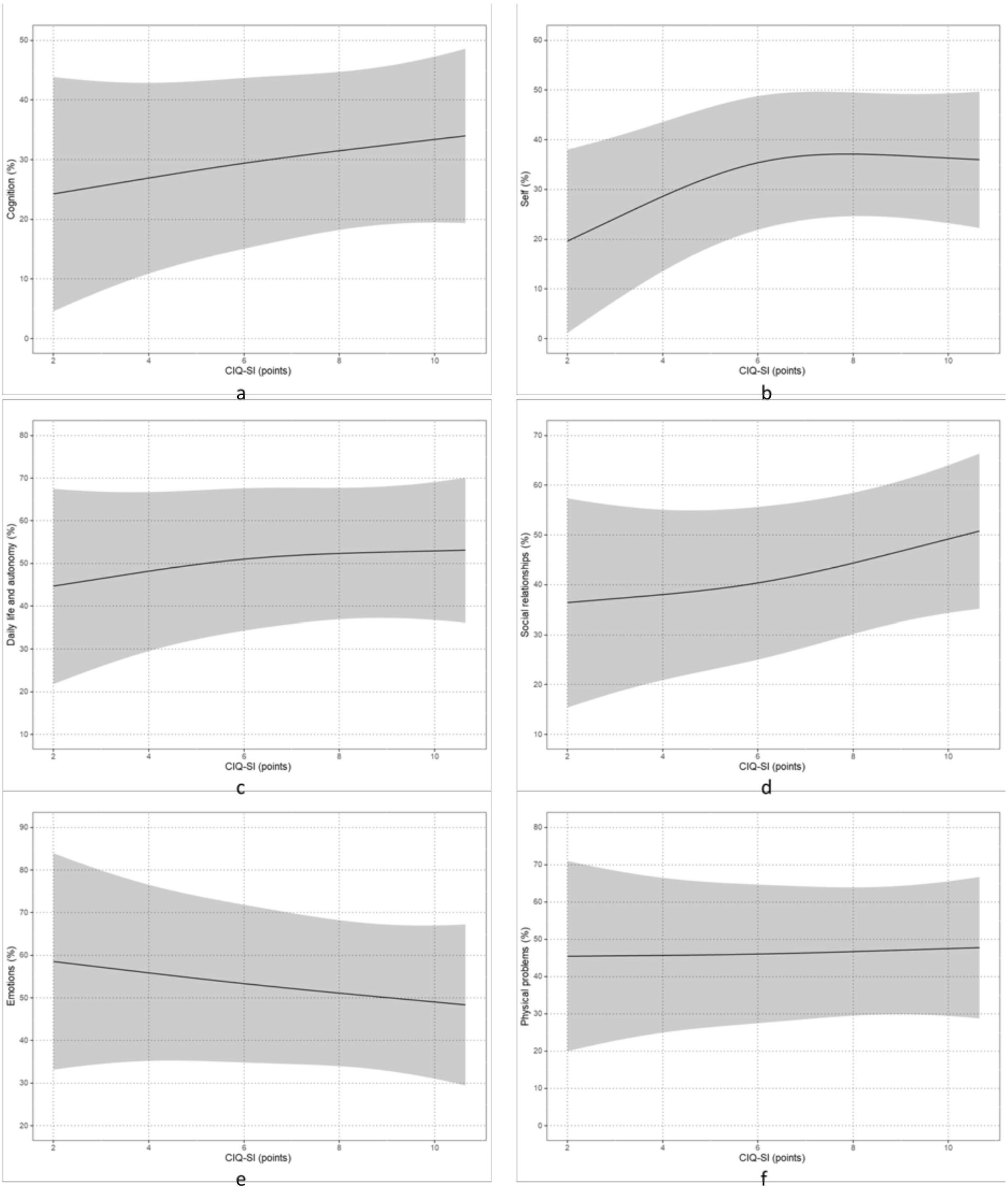

Figure 3: Relationship Between CIQ-SI and QOLIBRI Subscales

Nonlinear Regression Analysis: a) p=0.437 b) p=0.018 c) p=0.589 d) p=0.156 e) p=0.581 f) p=0.968

SI: Social Integration Scale

QOLIBRI: Quality of Life after Brain Injury

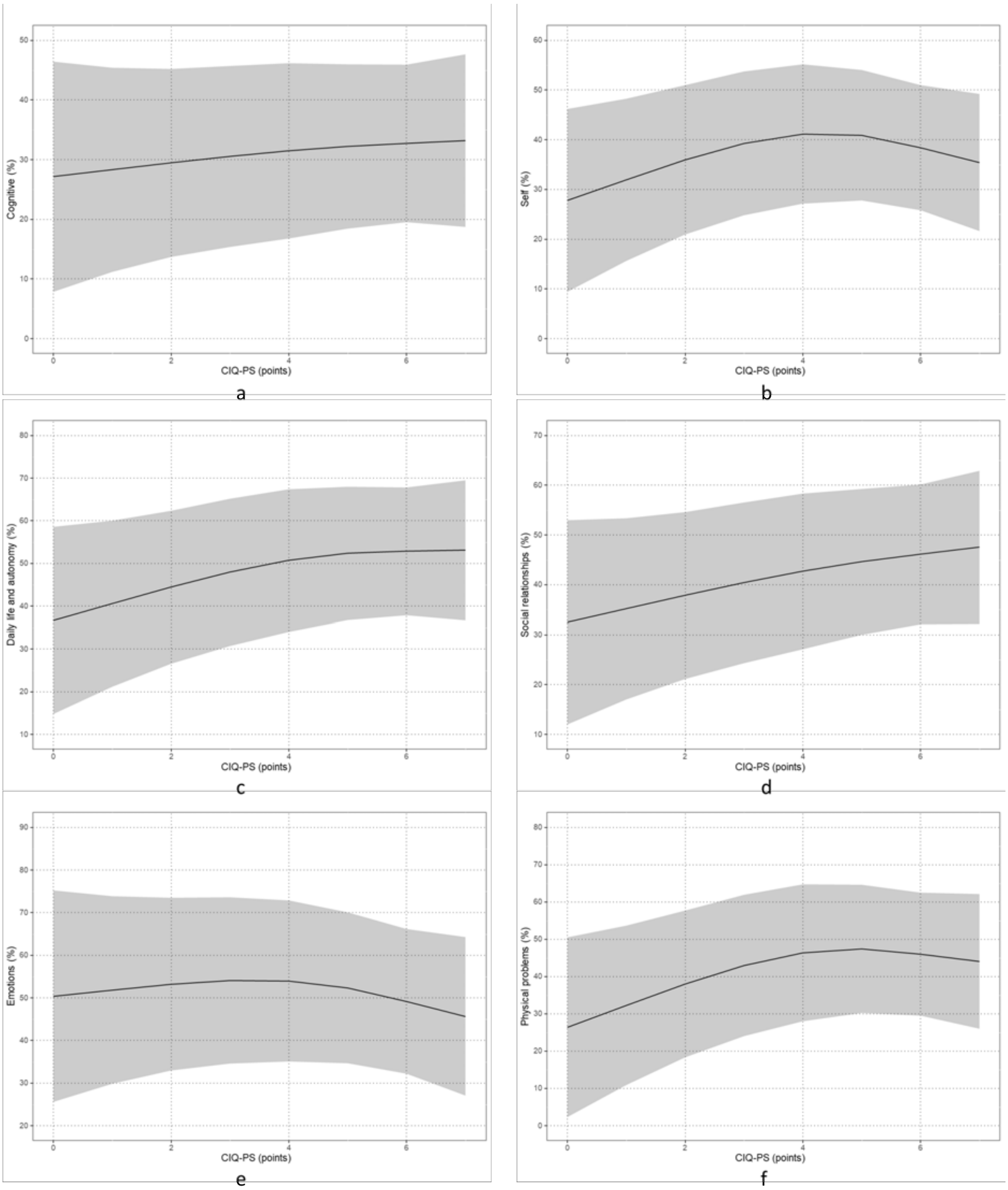

Figure 4: Relationship Between PS and QOLIBRI Subscales

Nonlinear Regression Analysis: a) p=0.707 b) p=0.128 c) p=0.119 d) p=0.159 e) p=0.579 f) p=0.062

PS: Productivity Scale

QOLIBRI: Quality of Life after Brain Injury
