# Supplementary material for: The relationship between social participation and quality of life in individuals with traumatic brain injury
Source: Fujita Med J. 2025 Apr 17;11(3):111–20. doi: 10.20407/fmj.2024-016 (PMC12327209; doi:10.20407/fmj.2024-016)
Supplement: Supplementary file 3 — Supplementary Tables [file fmj-11-111-s003.pdf]

Supplementary Tables

Table 1: CIQ Evaluation Items and Content

CIQ sub scale	content
Home integration	Shopping for necessities<br>Prepares meals<br>Housework<br>Caring for children<br>Social arrangements
Social integration	Personal finances<br>Frequency of shopping<br>Frequency of leisure activities<br>Frequency of visiting friends or relatives<br>Who do you participate in leisure activities with?<br>Having a best friend
Productivity	Travel outside of home<br>Work<br>School<br>Volunteer work

CIQ: Community Integration Questionnaire

Table 2: QOLIBRI Evaluation Items and Content

QOLIBRI sub scale	Content
Cognition	Cognitive problems such as memory, attention, expressive speech, and decision making
Self	Aspects of self, including energy, motivation, physical appearance, and self-esteem
Daily Life and autonomy	Independence, activities of daily life, and participation in social roles
Social relationships	Relationships with friends, family, and partner "Bothered" items
Emotions	Feelings of depression, anxiety, loneliness, boredom, and anger
Physical problems	Physical problems, such as slowness, pain, sensory impairment, or other consequences of injury

QOLIBRI: Quality of Life after Brain Injury
